# Unveiling the antibacterial potential of green-synthesized silver chloride and silver chromium nanoparticles from *Pelargonium graveolens* extract through molecular docking and bioactivity studies

**DOI:** 10.1128/spectrum.01142-25

**Published:** 2025-11-25

**Authors:** Mariam A. Ramadan, Zeinab Hachem, Alaa M. Abdallah, Nour El Ghouch, Ahmed F. El-Sayed, Mahmoud I. Khalil, Rana El Hajj

**Affiliations:** 1 Department of Biological Sciences, Faculty of Science, Beirut Arab University 67025, Beirut, Lebanon; 2 Department of Physics, Faculty of Science, Beirut Arab University 67025, Beirut, Lebanon; 3 Department of Chemistry, Faculty of Science, Beirut Arab University 67025, Beirut, Lebanon; 4 Microbial Genetics Department, Biotechnology Research Institute, National Research Centre 668878 , Giza, Egypt; 5 Egypt Center for Research and Regenerative Medicine (ECRRM) 594871, Cairo, Egypt; 6 Faculty of Science, King Salman International University 712685, Ras-Sedr, Egypt; 7 Molecular Biology Unit, Department of Zoology, Faculty of Science, Alexandria University 54562, Alexandria, Egypt; City of Hope Department of Pathology, Duarte, California, USA

**Keywords:** antibacterial activity, silver/silver chloride, silver chromium, nanoparticles, biofilm

## Abstract

Green nanotechnology exhibits significant biomedical applications. In this study, green-synthesized silver/silver chloride (Ag/AgCl) and silver chromium (AgCr) nanoparticles (NPs), using *Pelargonium graveolens* leaf extracts (PLE), were characterized using X-ray diffraction (XRD), scanning electron microscopy (SEM) with energy dispersive X-ray spectroscopy (EDX), X-ray photoelectron spectroscopy (XPS), Fourier transform infrared spectroscopy (FTIR), ultraviolet–visible absorption spectroscopy (UV-Vis), and photoluminescence spectroscopy (PL). The study examined the antimicrobial activities of PLE and NPs on various bacterial isolates. PLE was effective against Gram-positive bacteria, Ag/AgCl-NPs against resistance strains, AgCr-NPs had a MIC of 125 µg/mL, and MBC was reported against *K. pneumoniae*. The bactericidal activity of PLE exhibited best effect at 24 h, while NPs killing activity start from the second hour to reach best effect at 24 h. Biofilm formation was decreased in a dose-dependent manner. This study used molecular docking to evaluate the binding interactions of NPs with key antibacterial target proteins in four pathogenic bacteria: *A. baumannii*, *K. pneumoniae*, *Methicillin-resistant S. aureus* (MRSA), and *Methicillin-sensitive S. aureus* (MSSA). The target proteins included Penicillin-Binding Proteins (PBPs) in *A. baumannii* (−10.30 kcal/mol for AgCl-NPs, −10.55 kcal/mol for AgCr-NPs), KPC-2 carbapenemase in *K. pneumoniae* (−13.00 kcal/mol for AgCl-NPs, −14.50 kcal/mol for AgCr-NPs), PBP2a in MRSA (−12.60 kcal/mol for AgCl-NPs, −12.80 kcal/mol for AgCr-NPs), and PBP3 in MSSA (−8.50 kcal/mol for AgCl-NPs, −9.50 kcal/mol for AgCr-NPs). The results showed extensive hydrogen bonds and hydrophobic interactions, suggesting AgCl and AgCr NPs could inhibit these enzymes, disrupt bacterial cell wall synthesis, and overcome resistance mechanisms.

**IMPORTANCE:**

The growth of antibiotic resistance poses a huge threat to global public health, prompting researchers to search for novel approaches to combat this rising issue. Nanotechnology, or the manipulation of materials at the nanoscale, has emerged as a possible route for combating antibiotic resistance. The green-synthesized Ag/AgCl-NPs and AgCr-NPs demonstrated high antibacterial activities against several bacterial isolates. This study indicates that Ag/AgCl-NPs and AgCr-NPs can serve as a promising therapy to treat bacterial infections, especially those that are resistant to drugs.

## INTRODUCTION

Nanoscience is a rapidly emerging, interdisciplinary field with various applications in science and technology (1). This field focuses on the creation and application of nanostructured materials, which typically range in size from 1 to 100 nm. The rise of multidrug-resistant (MDR) bacteria and the shortage of effective drugs highlight the urgent need for novel antibiotics (2, 3). In this context, nanoparticles represent a viable alternative for managing bacterial illnesses, especially those triggered by multidrug-resistant organisms.

Several approaches have been developed for the synthesis of NPs, with each possessing distinct advantages and disadvantages. Nanoparticles can be synthesized using either physical or chemical processes (4). While these techniques can yield particles with the desired properties, they often involve high cost, significant effort, and potentially harmful to the environment and living organisms. To address these limitations, biological synthesis has emerged as a sustainable alternative, using diverse sources such as plant extracts and microbes (5).

Green-based synthesis of NPs is currently recognized as the gold standard method among biological approaches, primarily due to its simplicity and the variety of plant sources available. Plant extracts contain bioactive compounds such as proteins, flavonoids, terpenoids, ascorbic acid, and polyphenols, which not only exhibit natural antibacterial activity but also play critical roles in metal ion absorption, precursor salt reduction, and nanoparticle capping (6). This eco-friendly approach has gained broad acceptance in nanotechnology for producing safe nanoparticles (7). Silver nanoparticles (Ag-NPs) are widely used for physical, biological, and pharmacological applications (8). Their efficacy as anti-cancer and antibacterial agents has gained a lot of interest due to their broad spectrum of activity and high effectiveness. Additionally, chromium III oxide (Cr_2_O_3_) nanoparticles possess unique properties that support their use in science and technology, particularly in corrosion resistance, high-temperature stability, liquid crystal displays, green pigments, and catalysis (9–12).

Numerous researches on *Pelargonium graveolens* extracts have demonstrated their anti-microbial, anticancer, and cytotoxic effects (12–14). *P. graveolens* has been utilized in the management of various illnesses, including nephritis, wounds, sore throats, and inflammation (15–17).

The aim of this study was to synthesize Ag-NPs and Ag/Cr-NPs using *P. graveolens* leaf extract. The physiochemical characteristics of the synthesized NPs were assessed through characterization using XRD, SEM, EDX, XPS, FTIR, UV–Vis, and PL. Antibacterial and antibiofilm activities were evaluated against Gram-positive and Gram-negative bacteria, while molecular docking was performed to examine the interactions of Ag-NPs and Ag/Cr-NPs with key enzymatic targets of clinically relevant pathogens.

## RESULTS

### Characterization of Ag/AgCl-NPs and AgCr-NPs

#### XRD analysis

**Fig 1:**
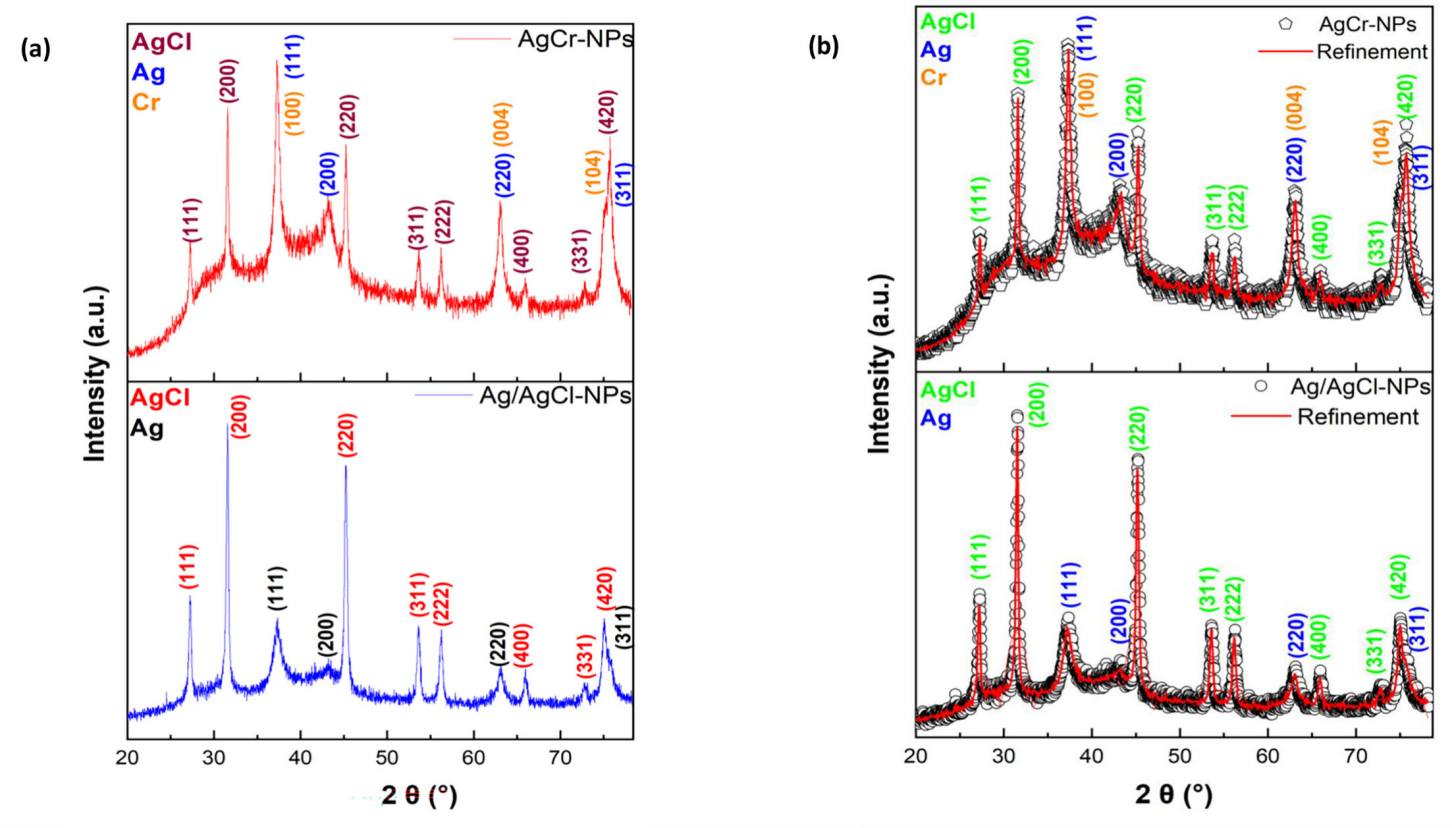
(**a**) The XRD patterns and (**b**) the Rietveld refinement of the XRD patterns of Ag/AgCl-NPs and AgCr-NPs.

The structural properties of the synthesized NPs were investigated through XRD analysis, as shown in Fig. 1a and b for Ag/AgCl-NPs and AgCr-NPs. Sharp peaks at 2θ around 38.1°, 44.3°, 64.4°, and 77.4° correspond to the (111), (200), (220), and (311) planes of Ag-NPs, confirming their formation. In Ag/AgCl-NPs, additional peaks at 27.8°, 32.2°, 46.2°, 54.8°, 57.4°, 67.4°, 74.4°, and 76.7° correspond to AgCl planes (111), (200), (220), (311), (222), (400), (331), and (420), confirming AgCl-NPs formation.

For AgCr-NPs, diffraction peaks at 37.3°, 62.9°, and 75.5° are assigned to the (100), (004), and (104) planes of Cr. Ag-related peaks appear at 37.3° (111), 43.3° (200), 64.4° (220), and 77.4° (311), while AgCl peaks are identified at 27.2° (111), 31.5° (200), 45.2° (220), 53.6° (311), 57.4° (222), 67.4° (400), 74.4° (331), and 76.7° (420).

Rietveld refinement using the MAUD program revealed a lattice parameter *a* = 4.16 Å and a crystallite size of 6.5 nm for Ag/AgCl-NPs. For AgCr-NPs, the crystallite size was determined via Scherrer’s formula: *D*$=\frac{0.9\lambda }{\beta cos\theta }$ , where *D* is the crystallite size, *λ* = 1.5406 Å is the wavelength of X-ray, *b* is the full width half maximum (FWHM) of the peak in radians, and *θ* is the Bragg angle. The calculated crystallite size for AgCr-NPs was 7.9 nm, and the lattice parameters were found to be *a* = 2.77 Å and *c* = 5.90 Å.

#### SEM-EDX analysis

**Fig 2:**
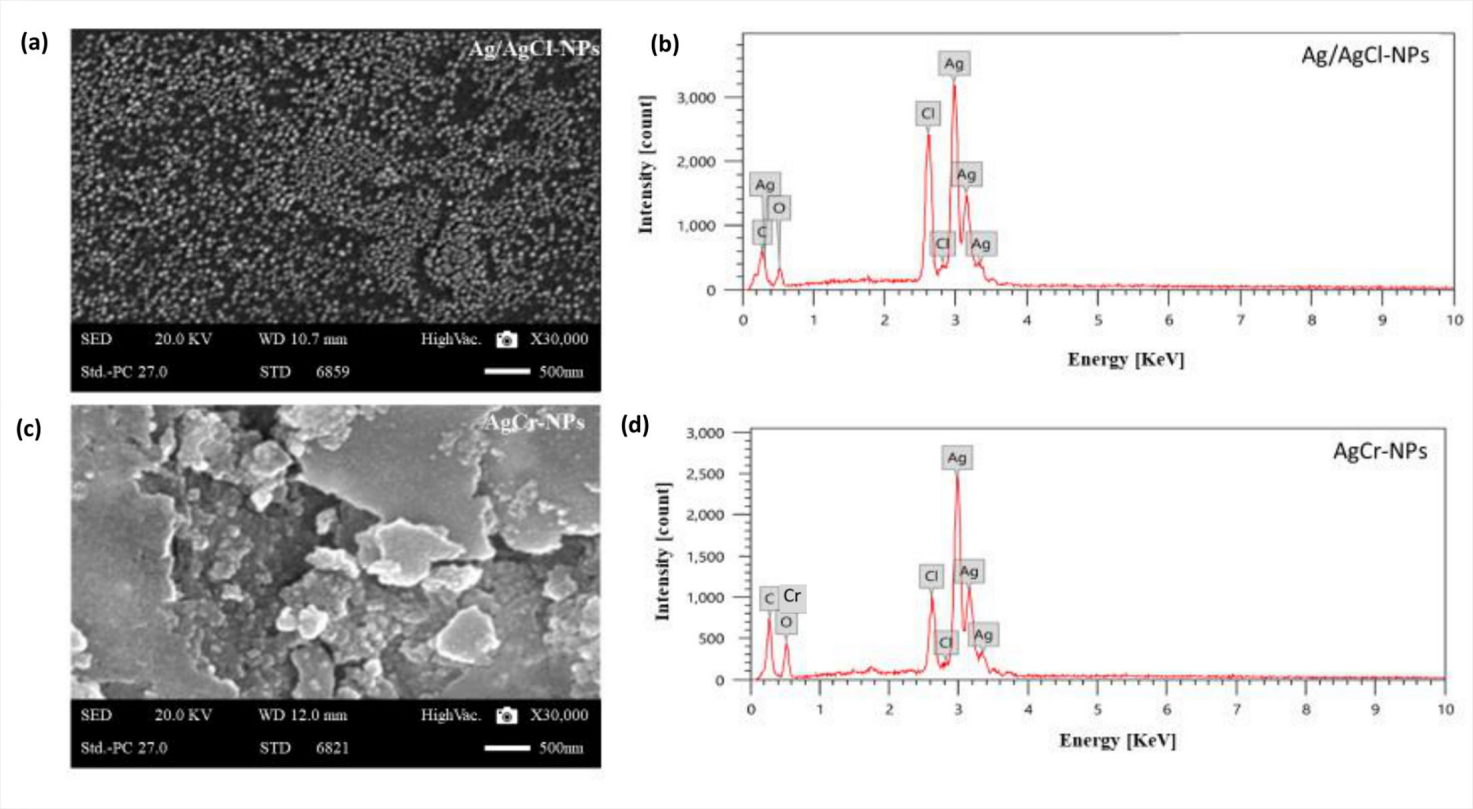
The SEM micrograph (**a and c**), and the EDX spectrum (**b and d**), of the green synthesized Ag/AgCl-NPs and AgCr-NPs, using PLE.

The SEM micrograph revealed that Ag/AgCl-NPs were spherical, non-uniformly distributed, with particle sizes ranging from 20.98 to 23.75 nm (Fig. 2a). Furthermore, AgCr-NPs were also spherical, with sizes ranging from 13 to 17 nm (Fig. 2c). Elemental composition was confirmed by EDX analysis. For Ag/AgCl-NPs, EDX spectra showed the existence of Ag (29.8%), C (10%), O (40.6%), and Cl (19.6%) (Fig. 2b). For AgCr-NPs, EDX spectra indicated that the NPs consisting of C (45.0%), O (38.2%), Cl (4.0%), and Ag (12.8%) (Fig. 2d), with Cr detected in trace amounts. EDX analysis showed high emission energy at 3 keV, confirming the formation of Ag-NPs (Fig. 2b).

#### XPS analysis

**Fig 3:**
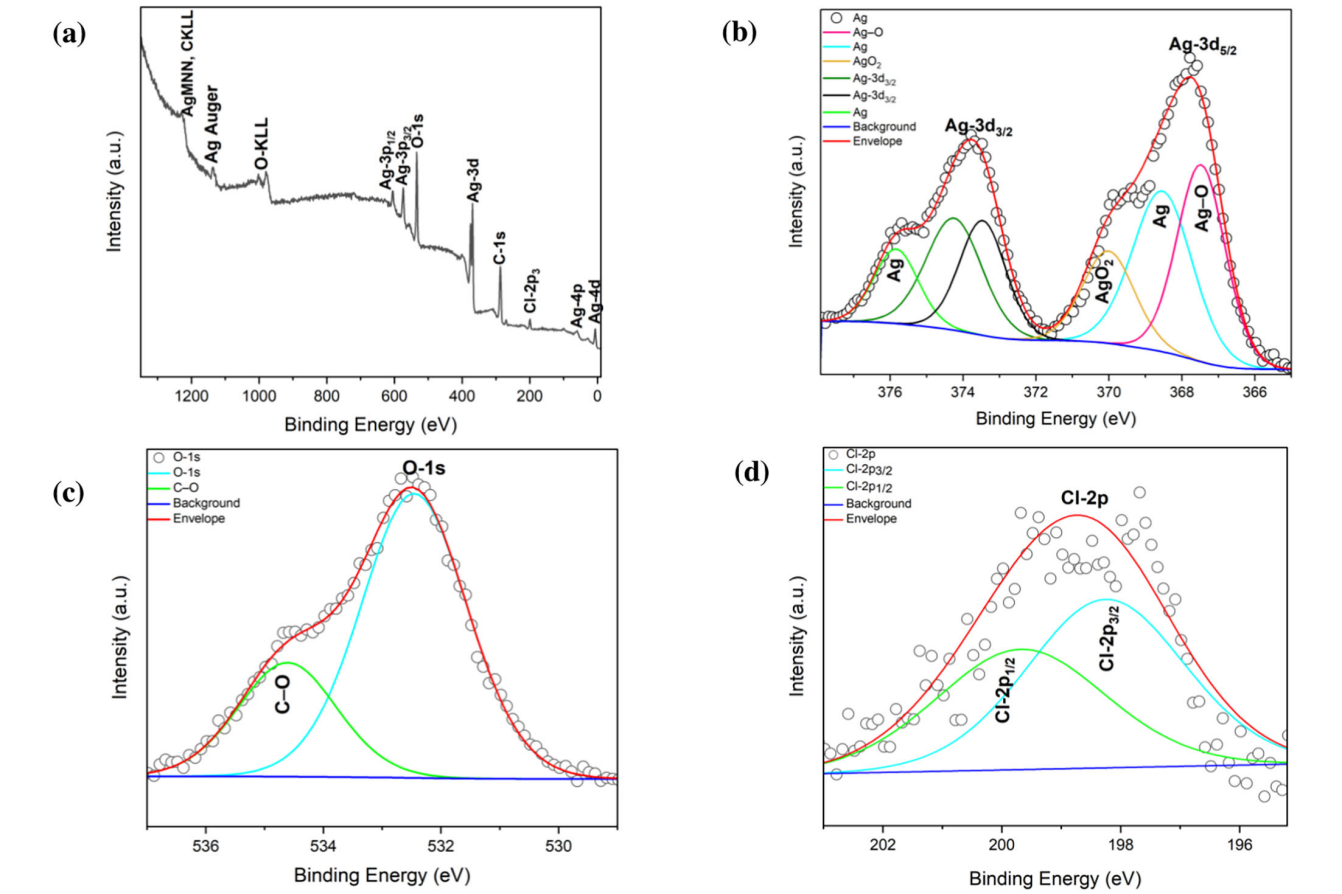
The XPS spectra of the green synthesized Ag/AgCl (**a**). Deconvolution of the main spectra lines of (**b**) Ag-3d, (**c**) O-1s, and (**d**) Cl-2p.

**Fig 4:**
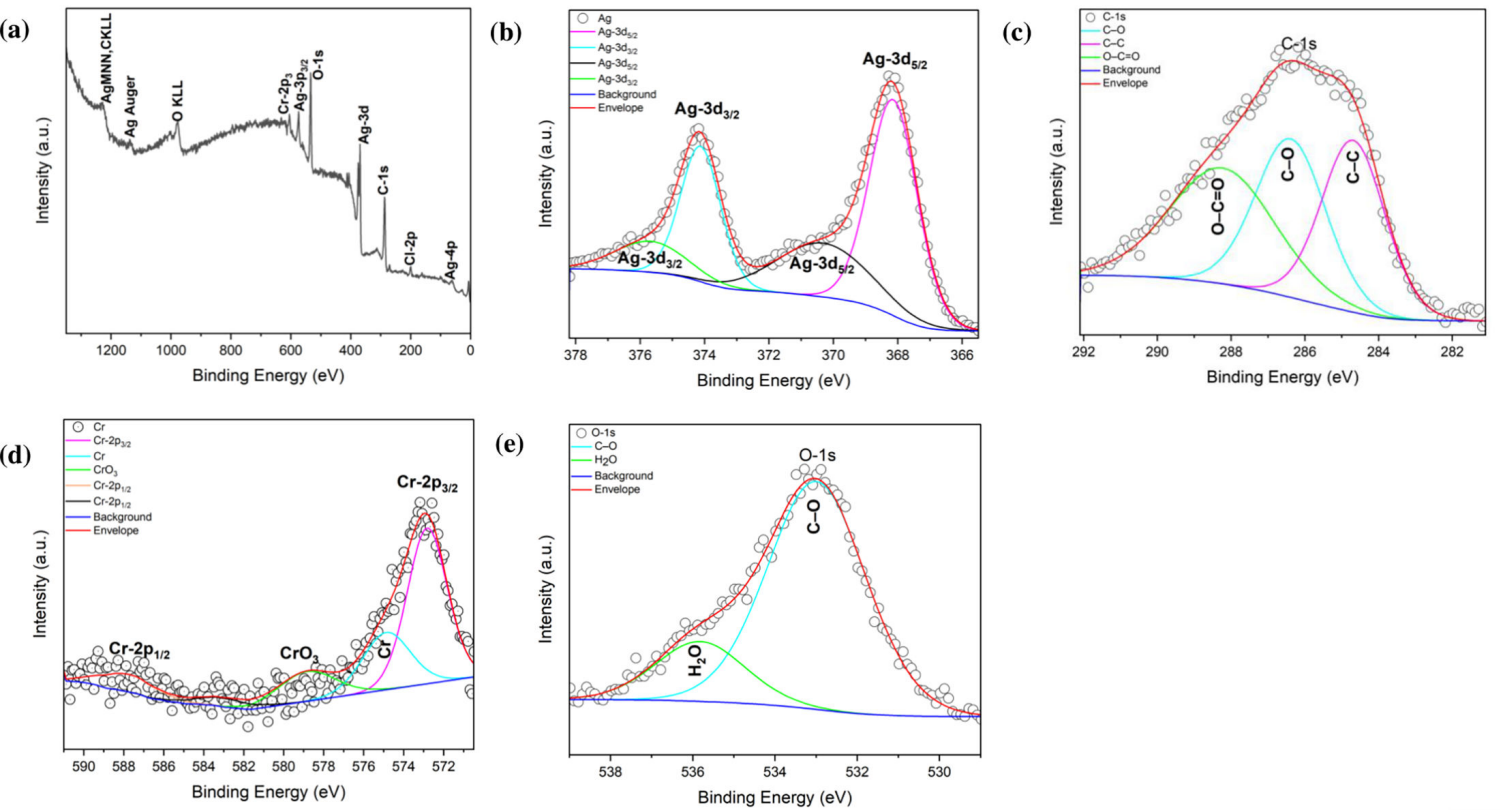
The XPS spectra of the green synthesized AgCr-NPs (**a**). Deconvolution of the main spectra lines of (**b**) Ag-3d, (**c**) C-1s, (**d**) Cr-2p, and (**e**) O-1s.

The chemical state and near-surface composition of Ag/AgCl-NPs and AgCr-NPs were determined using XPS. The XPS spectrum of Ag/AgCl-NPs shows the presence of Carbon C-1s, silver Ag-3d, oxygen O-1s, and Chloride Cl-2p_3/2_ (Fig. 3a). High-resolution XPS (HR-XPS) of binding energy (BE) linked to the Ag-3d area showed Ag-3d_5/2_ and Ag-3d_3/2_ peaks at 367.7 and 373.7 eV, respectively (Fig. 3b) (18). Additional peaks at 368.5, and 367.4 eV indicating the formation of Ag and AgO, while deconvolution of the Ag-3d3/2 region revealed peaks at 376 and 370.5 eV, confirming the existence of Ag and AgO_2_, respectively (18, 19). The O-1s HR-XPS spectrum showed a peak at 532.4 eV attributed to the Ag–O bond, and a peak at 534.5 eV corresponding to C–O bonds (Fig. 3c) (19, 20). The Cl-2p region exhibited peaks at 198.8 eV (Cl-2p_3/2_) and 199.8 eV (Cl-2p_1/2_) (21). The C-1s peak at 287.6 eV was assigned to C–C or C–H vibrations (Fig. 3d) (22). The XPS survey spectrum of AgCr-NPs displayed Ag3d_3/2_ and Ag3d_5/2_ peaks split into doublets in the ranges of 368.1–370.3 and 374.1–375.8 eV, respectively (Fig. 4a and b) (23). The C-1s XPS spectrum showed peaks at BE of 284.6, 286.3, and 288.3 eV, associated with C-C, C-O, and –C = O, respectively (Fig. 4c) (24). HR-XPS of Cr-2p revealed peaks at 587.5 eV (Cr-2p_1/2_) and 572.8 eV (Cr-2p_3/2_), with additional peaks at 578.7 eV and 574.8 eV attributed to Cr₂O₃ (major phase) and Cr(OH)₃ (minor phase) (Fig. 4d) (25, 26). The O-1s XPS spectrum showed O-1s binding energies at 533.0 eV, indicating the presence of the C–O bonds and another at 535.3 eV, likely due to environmental contamination or H₂O (Fig. 4e) (27, 28).

#### FTIR analysis

**Fig 5:**
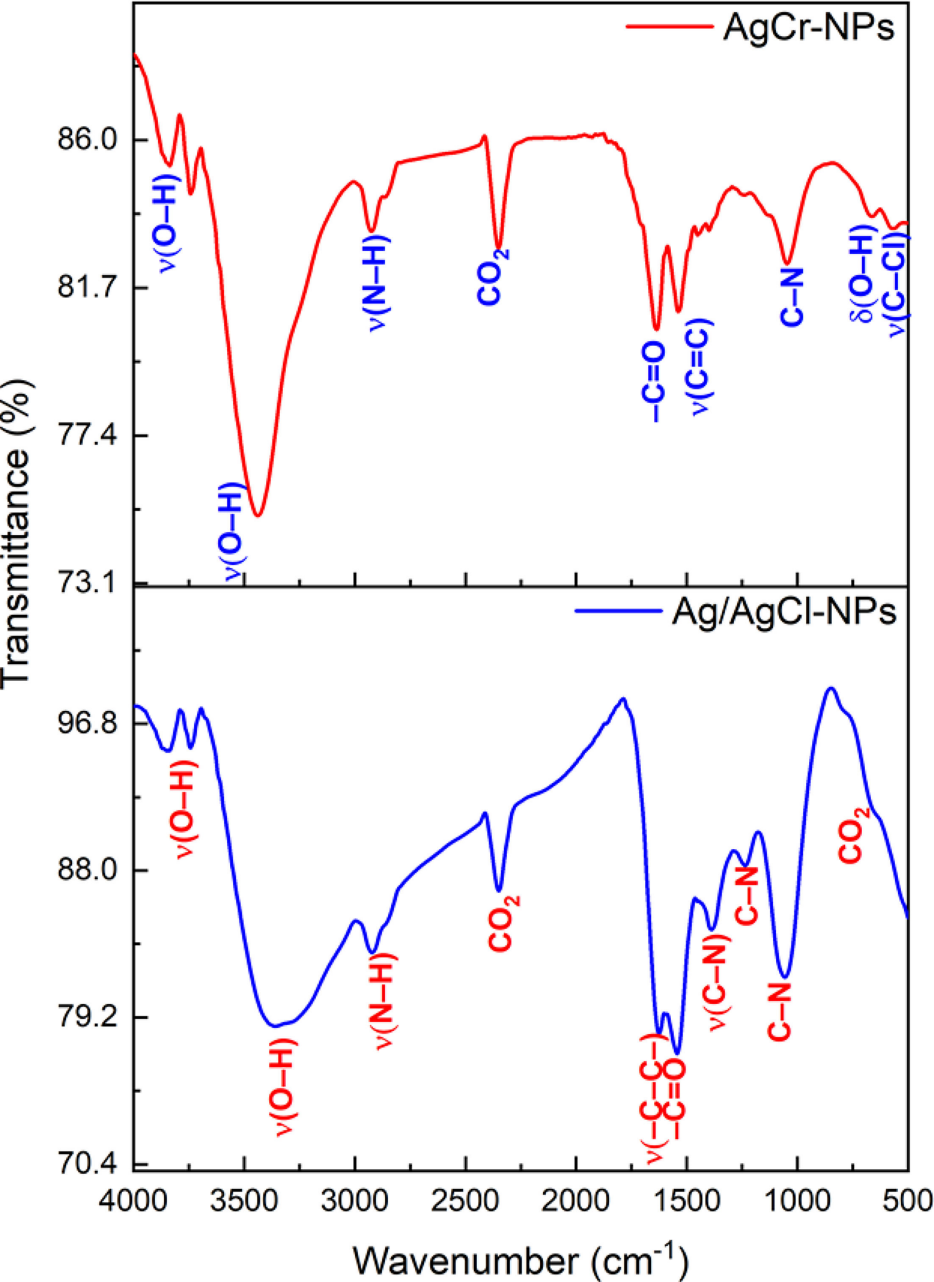
FTIR spectra of the green synthesized Ag/AgCl-NPs and AgCr-NPs.

The various functional groups in Ag/AgCl-NPs and AgCr-NPs were identified using FTIR measurements (Fig. 5). The bands at 3,000–3,800 cm^-1^ were the characteristic O–H stretching vibration (29). The bands within 3,500–2,800 cm^-1^ were assigned for N-H stretching vibration (30). The bands at 2,340.40 and 674.26 cm^1^ in Ag/AgCl-NPs and 2,368.73 cm^-1^ in AgCr-NPs are connected to alkene and atmospheric CO_2_ present in the instrument, respectively (31, 32). The peaks at 1,550.65 cm^-1^ in Ag/AgCl-NPs and 1,633.96 cm^-1^ in AgCr-NPs were linked to the –C = O group and the amide I band of protein, respectively (33). The stretching vibrations –C–C– in Ag/AgCl-NPs (aromatic) were known to be associated with an absorbance band detected at 1,621.29 cm^−1^ (34). The band at 1,537.11 cm^-1^ in AgCr-NPs was assigned to C = C stretching of aromatic compound (35). The bands between 1,023 and 1,087 cm^−1^ in Ag/AgCl-NPs and AgCr-NPs were assigned for C–N (aromatic and aliphatic amines), respectively (36). In Ag/AgCl-NPs, the peaks at 1,383 cm^−1^ correspond to C–N stretching of amines (36, 37). The stretching and bending vibration of O–H, which came from water molecules presented on the surface of prepared materials, is shown by the absorption peak situated approximately at 671.05 cm^−1^ (38). The band seen at 577.95 cm^−1^ belongs to the alkyl group’s C–Cl stretching (39).

#### UV spectrophotometry study

**Fig 6:**
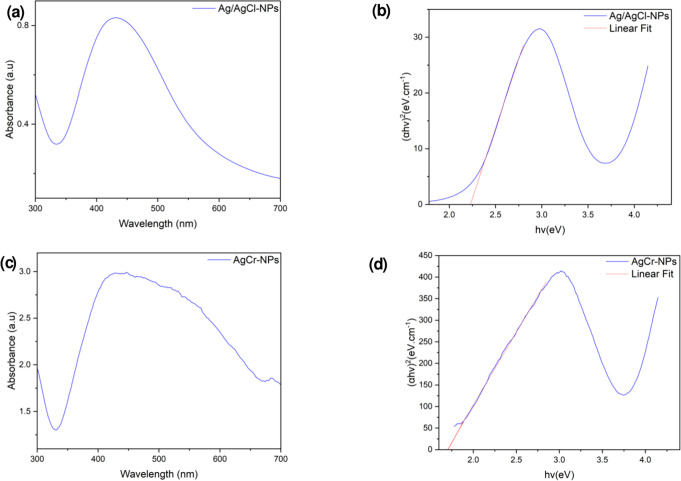
UV-visible spectrum (**a and c**), and Band gap energy graph (Tauc's plot) (**b and d**) of Ag/AgCl-NPs and AgCr-NPs.

The synthesis of Ag/AgCl-NPs and AgCr-NPs in aqueous solution was monitored by measuring the absorption spectra at 300–700 nm. A single, strong, and broad surface plasmon resonance (SPR) peak was found at 428 nm, confirming the synthesis of Ag/AgCl-NPs (Fig. 6a). Moreover, an absorption peak at 430 nm in the UV-vis spectrum confirms the synthesis of AgCr-NPs (Fig. 6c). The energy band gap can be determined from the Tauc plot between (αhv)^2^ vs energy of photon (hv). The intercept of the tangent on the Tauc plot results in a direct band gap. The band gap energy for Ag/AgCl-NPs and AgCr-NP was found to be 2.23 eV and 1.70 eV, respectively (Fig. 6c and d).

#### PL studies

**Fig 7:**
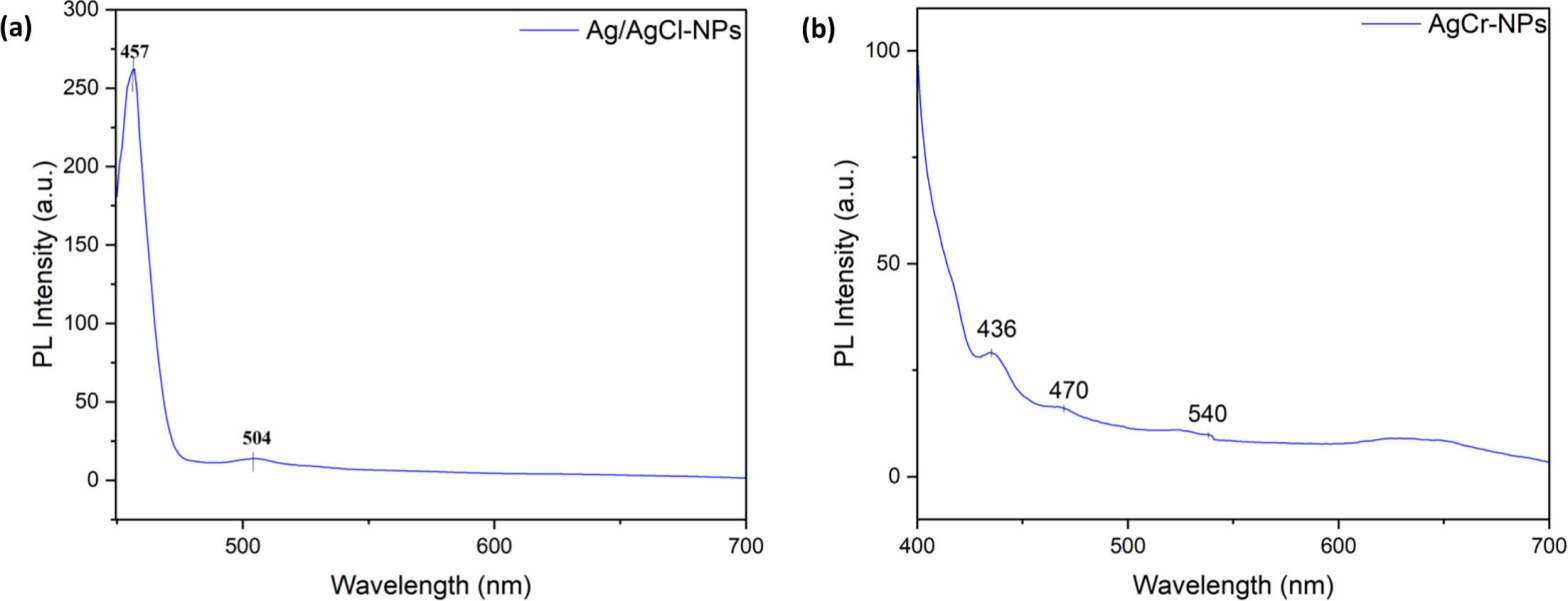
Photoluminescence spectra of Ag/AgCl-NPs (**a**) and AgCr-NPs (**b**) synthesized from PLE.

The PL spectra obtained from the synthesized Ag/AgCl-NPs and AgCr-NPs were shown in Fig. 7a and b. PL emission of nanoparticles was measured in the visible region (400–700 nm). Ag/AgCl-NPs showed a primary emission band which is a superimposition of two peaks, one green luminescence at 504 nm and one blue emission at 457 nm, respectively (Fig. 7a). With Cr doping, the green band shifted to 540 nm, and two blue bands at 470 and 436 nm appeared (Fig. 7b).

### Qualitative phytochemical screening

The main metabolites of aqueous extract of PLE were assessed qualitatively. The aqueous extract was found to contain flavonoids, phenols, terpenoids, and steroids; however, anthraquinones and saponins were not identified (Table 1).

**TABLE 1:** Phytochemical analysis conducted on aqueous extract of PLE

Phytochemicals	(+: Presence/−: Absence)
*Flavonoids*	+
*Phenolics*	+
*Saponins*	−
*Terpenoids*	+
*Steroids*	+
*Anthraquinone*	−

### Antibacterial effect of green synthesized Ag/AgCl-NPs and AgCr-NPs

#### Determination of MIC and MBC

The results of MIC and MBC were shown in Table 2. The MIC of PLE toward MSSA and MRSA was 500 μg/mL, while the MIC for *K. pneumoniae* and *A. baumannii* was found to be 1,000 μg/mL. However, PLE did not show MBC. The MIC for Ag/AgCl-NPs against MSSA was 250 μg/mL, while for MRSA, it was 125 μg/mL. For *K. pneumoniae,* it was 250 μg/mL, whereas for *A. baumannii,* it was 125 μg/mL. The MBC was reported in Ag/AgCl-NPs against *K. pneumoniae* at 1,000 μg/mL. AgCr-NPs exhibited an MIC value of 125 μg/mL against MSSA*,* MRSA, *K. pneumoniae*, and *A. baumannii*. The MBC of AgCr-NPs was not identified.

**TABLE 2:** MIC and MBC results of PLE, Ag/AgCl-NPs, and AgCr-NPs

Bacterial isolates	MSSA		MRSA		*K. pneumoniae*		*A.baumannii*	
	MIC	MBC	MIC	MBC	MIC	MBC	MIC	MBC
PLE	500 ± 0.013	>1,000	500 ± 0.026	>1,000	1,000 ± 0.010	>1,000	1,000 ± 0.015	>1,000
Ag/AgCl-NPs	250 ± 0.088	>1,000	125 ± 0.016	>1,000	250 ± 0.025	1,000	125 ± 0.006	>1,000
AgCr-NPs	125 ± 0.023	>1,000	125 ± 0.045	>1,000	125 ± 0.026	>1,000	125 ± 0.011	>1,000

#### Time-killing kinetics

**Fig 8:**
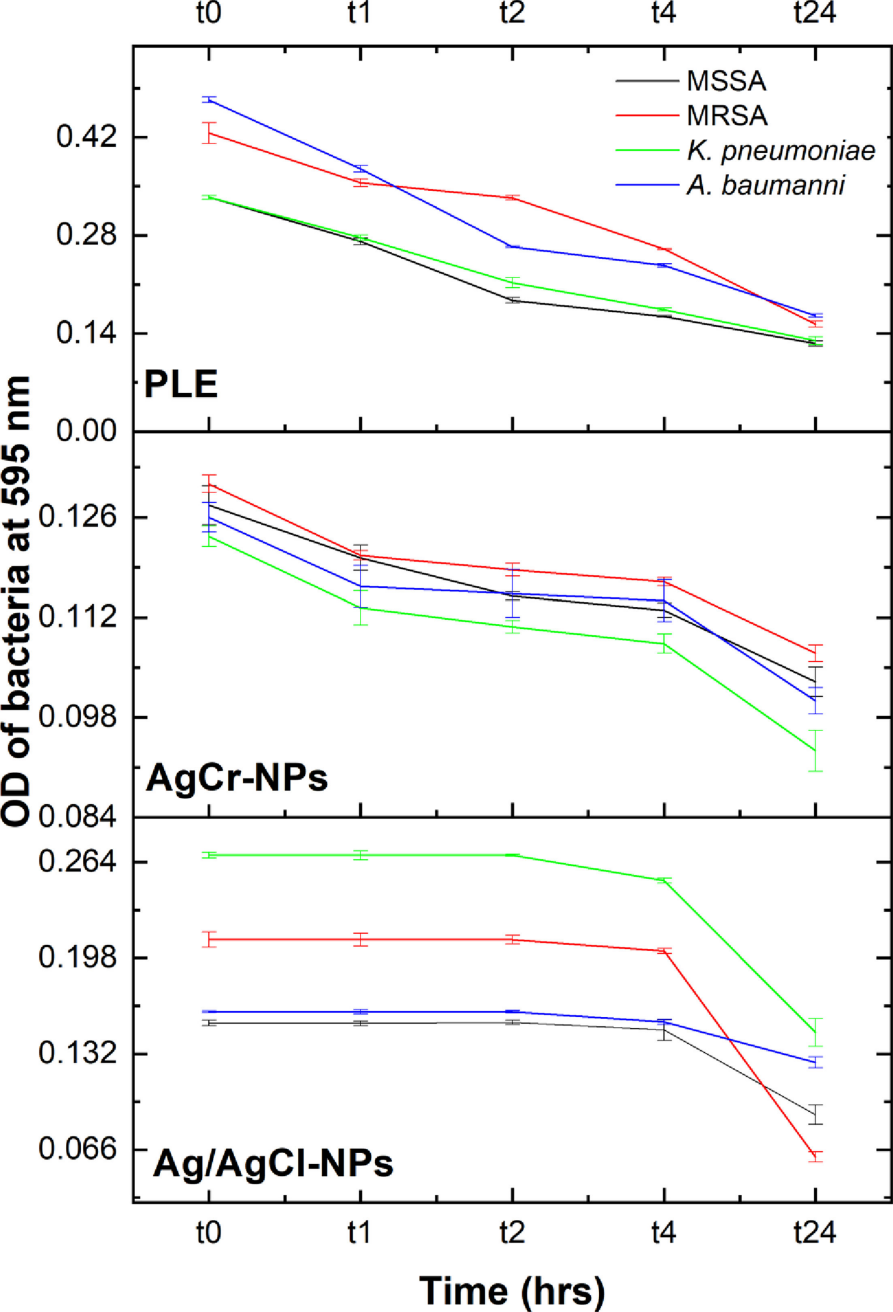
Time-kill curve assay for the effect of tested compound Ag/AgCl-NPs, AgCr- NPs, and PLE on four bacterial isolates.

A time-kill curve test was conducted to evaluate the antimicrobial efficacy (Fig. 8). AgCr-NPs and PLE significantly reduced bacterial growth within the first hour, with maximum inhibitory effects observed after 24 h. In contrast, Ag/AgCl-NPs exhibited a delayed bacteriostatic effect, beginning at 4 h, and achieving significant inhibition by 24 h.

#### Anti-biofilm activity of Ag/AgCl-NPs and AgCr-NPs

**Fig 9:**
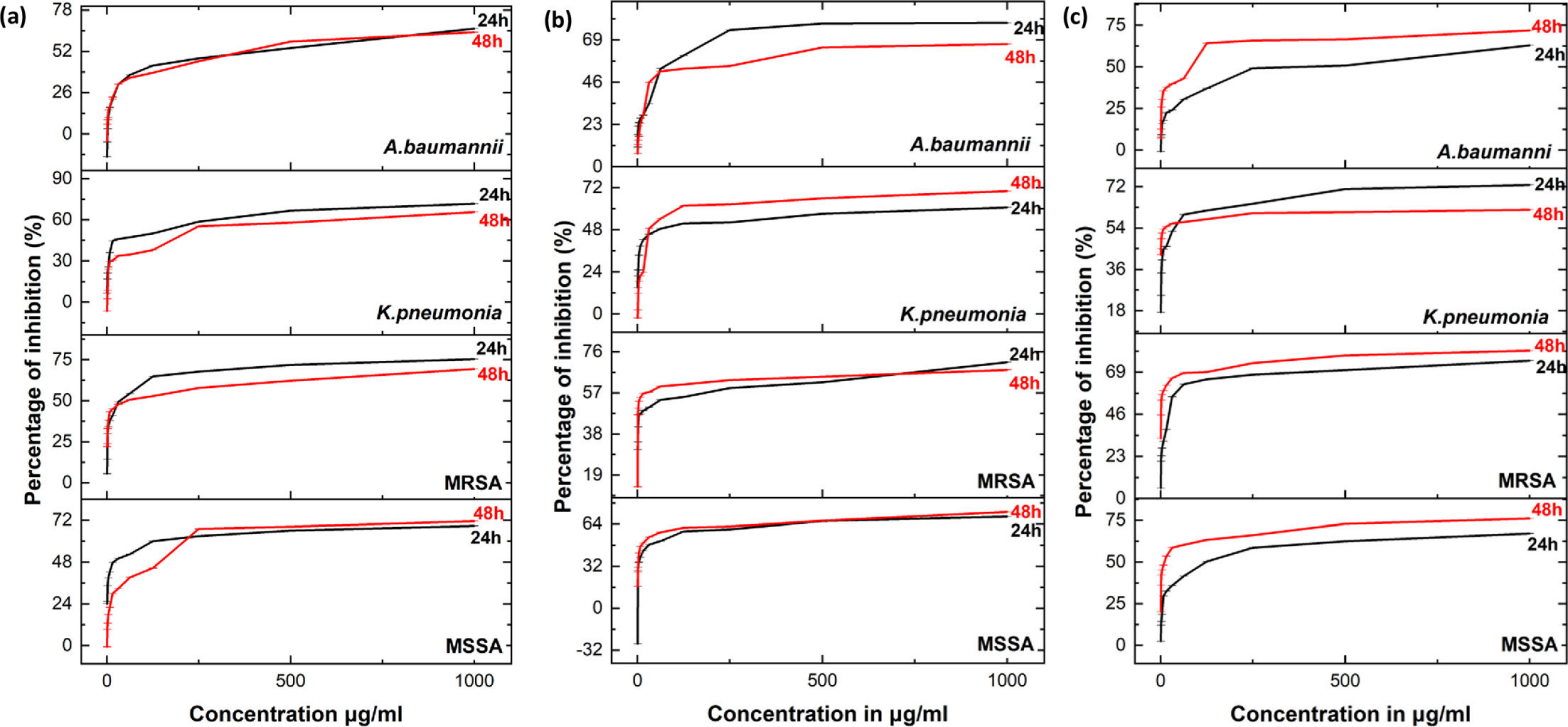
Effect of Ag/AgC1-NPs (**a**), AgCr-NPs (**b**), and PLE (**c**) on biofilm inhibition at 24 and 48 h.

**Fig 10:**
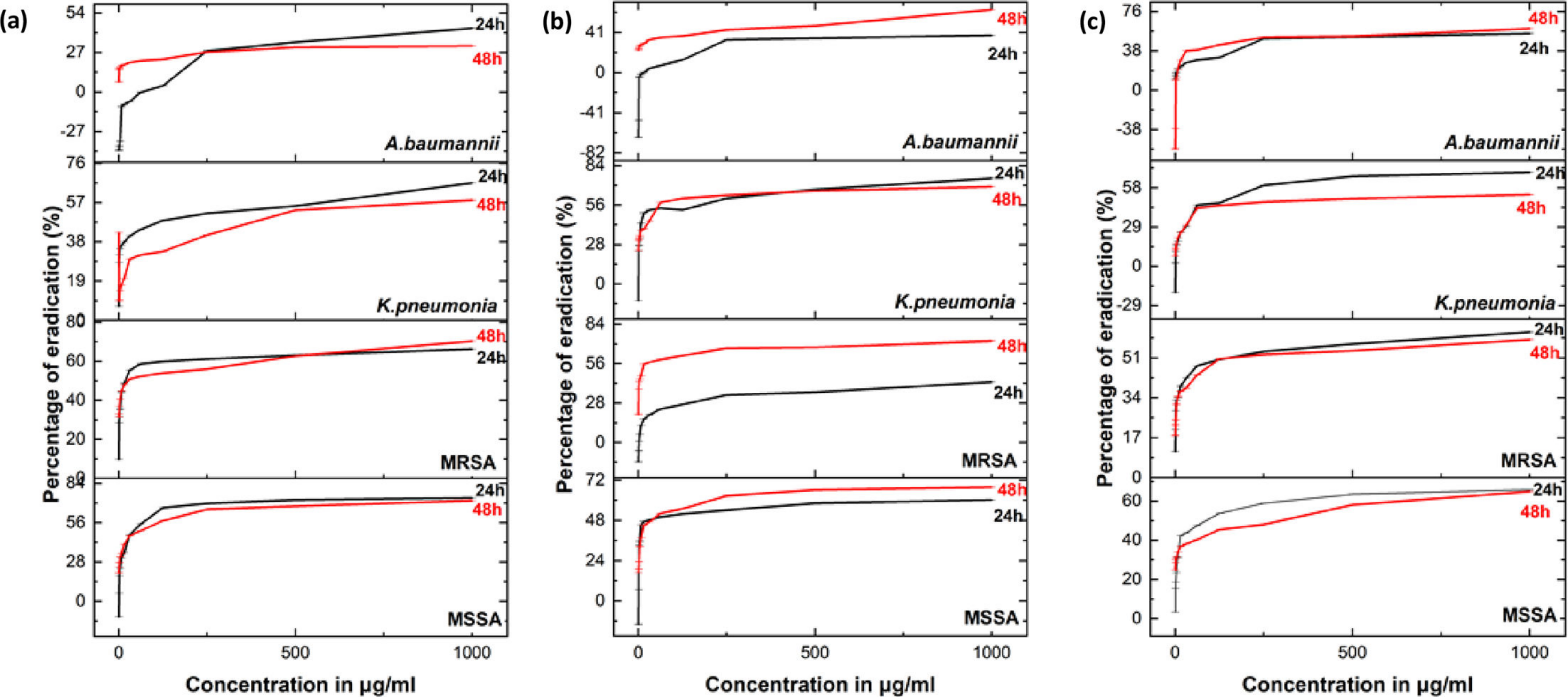
Effect of Ag/AgCl-NPs (**a**), AgCr-NPs (**b**), and PLE (**c**) on biofilm eradication at 24 and 48 h.

The anti-biofilm efficacy of PLE, Ag/AgCl-NPs, and AgCr-NPs was tested against MSSA, MRSA, *K. pneumoniae*, and *A. baumannii*. Our findings demonstrated that PLE, Ag/AgCl-NPs, and AgCr-NPs exerted significant, concentration-dependent inhibition of biofilm formation, as confirmed via the crystal violet assay (Fig. 9 and 10). PLE showed the highest inhibition (up to 75%). Ag/AgCl-NPs inhibited biofilms by 43%–67%, while AgCr-NPs showed inhibition in the range of 48%–60%. PLE, Ag/AgCl-NPs, and AgCr-NPs exhibited better biofilm inhibition compared to doxycycline against MSSA and *K. pneumoniae* at both 24 and 48 h. Additionally, AgCr-NPs proved to be more effective than doxycycline against MRSA and *A. baumannii*.

### Molecular docking of Ag/AgCl-NPs and AgCr-NPs with antibacterial target proteins

**Fig 11:**
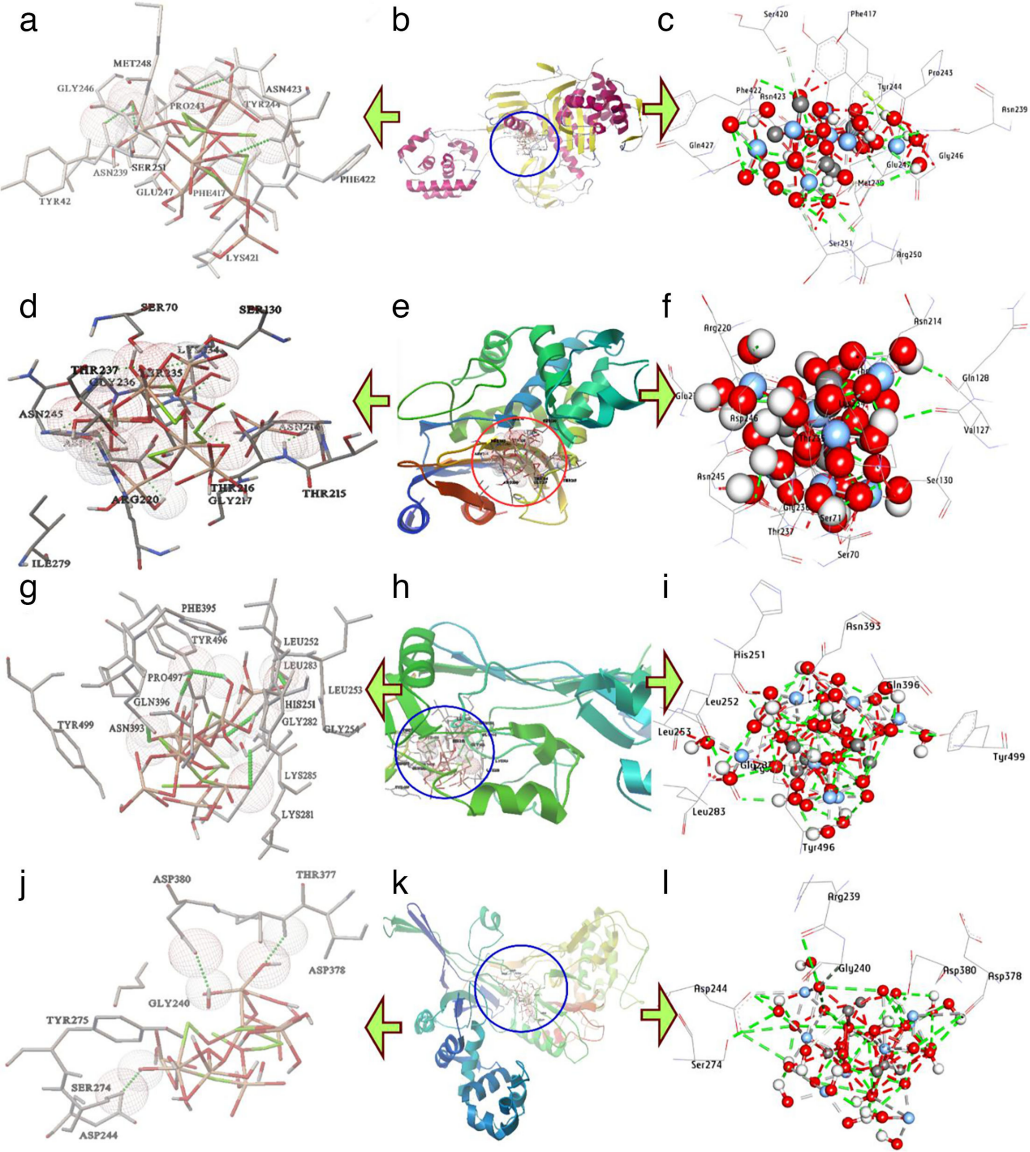
3D interactions of AgC1-NPs conformations at the binding pocket: (**a, b, and c**) penicillin-binding proteins of *A. baumannii* (PDB: ID 3UE1), (**d, e, and f**) KPC-2 Carbapenemase of *K. pneumoniae* (PDB: ID 20V5), (**g, h, and i**) Penicillin-Binding Proteins (PBP2a) of Methicillin-resistant *S. aureus* (PDB: ID 4CJN), and (**j, k, and l**) Penicillin-Binding Protein 3 (PBP3) of Methicillin-sensitive *S. aureus* (PDB: ID 3VSL).

**Fig 12:**
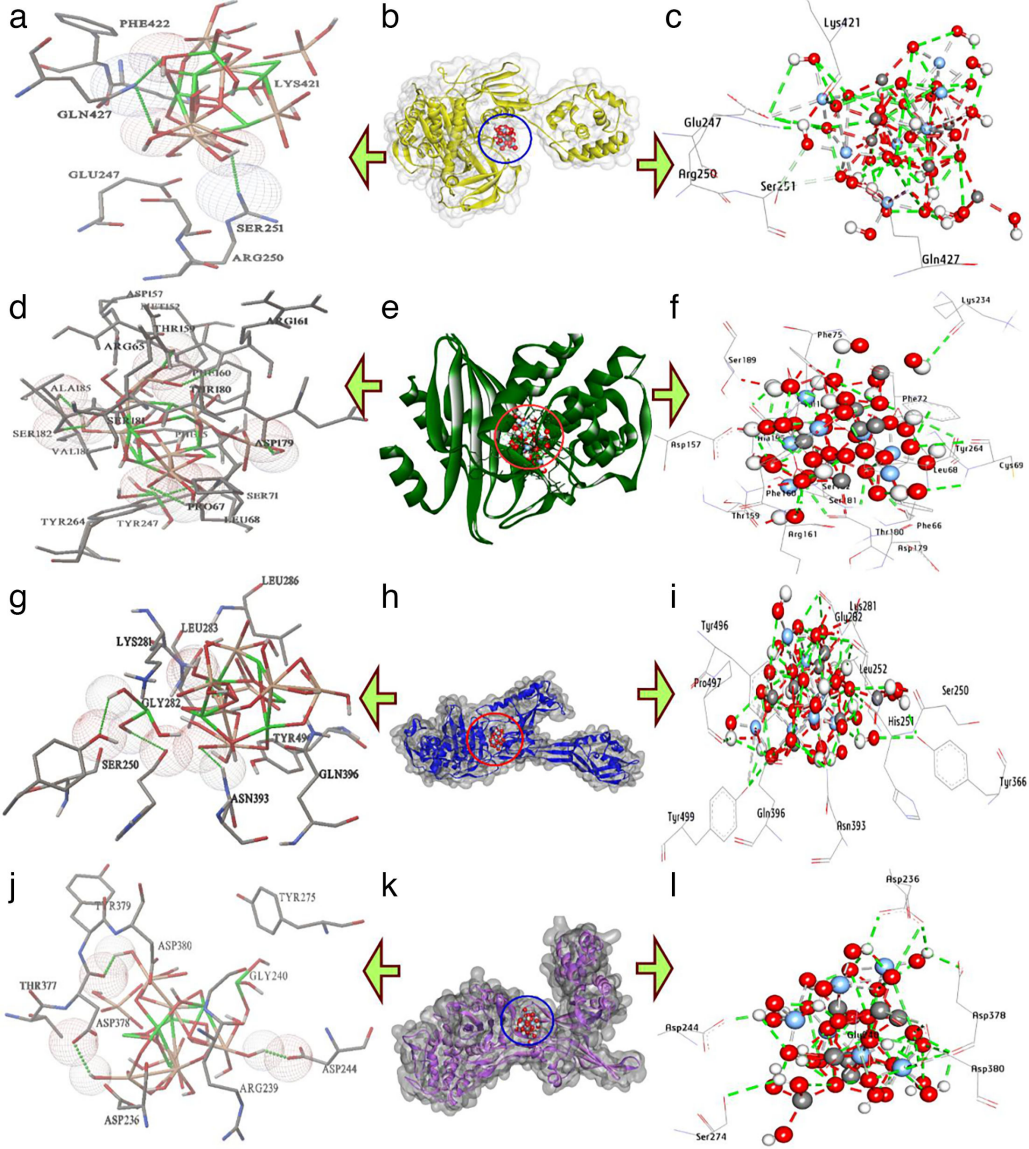
3D interactions of AgCr-NPs conformations at the binding pocket: (**a, b, and c**) penicillin-binding proteins of *A. baumannii* (PDB: ID 3UE1), (**d, e, and f**) KPC-2 Carbapenemase of *K. pneumoniae* (PDB: ID 20V5), (**g, h and i**) Penicillin-Binding Proteins (PBP2a) of Methicillin-resistant *S. aureus* (PDB: ID 4CJN), and (**j, k, and l**) Penicillin-Binding Protein 3 (PBP3) of Methicillin-sensitive *S. aureus* (PDB: ID 3VSL).

Penicillin-binding proteins (PBPs), essential enzymes for bacterial cell wall synthesis, serve as primary targets for beta-lactam antibiotics. Molecular docking analysis (Table 3) revealed that AgCl-NPs exhibited a binding affinity of −10.30 kcal/mol toward *A. baumannii* PBPs, while AgCr-NPs demonstrated a marginally stronger interaction at −10.55 kcal/mol. AgCl-NPs formed seven hydrogen bonds with residues Asn239, Met248, Arg250, Ser251, Asn423, Gln247, and Phe422 (Fig. 11a through c), along with five hydrophobic interactions (carbon-hydrogen bonds: Ser251, Ser420; metal-acceptor bond: Glu247; Pi-lone pair bond: Asp73). In contrast, AgCr-NPs established seven hydrogen bonds with Arg250, Gln427, Glu247, Lys421, and Gln247 (repeated with Gln427) and two hydrophobic interactions (carbon-hydrogen bond: Ser251; metal-acceptor bond: Glu247). Notably, shared residues in the catalytic pocket—Glu247, Gln427, Lys421, and Ser251 contributed to enhanced binding stability (Fig. 12a through c).

**TABLE 3:** Molecular interactions of AgCl-NPs with amino acids of the list of targets^*a*^

	Proteins	Hydrophilic interactions		Hydrophobic contacts		No. of H-Bonds	No. of total bonds	Affinity kcal mol−1
		Residue (H- Bond)	Length	Residue (bond type)	Length			
1	*A. baumannii*	Asn239, (H- Bond) Met248, (H- Bond) Arg250, (H- Bond) Ser251, (H- Bond) Asn423, (H- Bond) Gln247, (H- Bond) Phe422, (H- Bond)	2.20 2.71 2.49 2.51 2.18 2.29 2.89	Ser251, (C-H-Bond) Ser420, (C-H-Bond) Glu247, (Metal-Acceptor) Asp73 (Pi-Lone Pair)	3.05 3.54 2.44 2.69	7	11	−10.30
2	*K. pneumoniae*	Ser130, (H- Bond) Asn214, (H- Bond) Thr216, (H- Bond) Arg220, (H- Bond) Lys234, (H- Bond) Thr235, (H- Bond) Thr237, (H- Bond) Glu276, (H- Bond) Asp246, (H- Bond) Val127, (H- Bond) Gln128, (H- Bond) Asn245, (H- Bond)	1.97 2.77 2.32 1.97 1.74 2.35 2.88 3.02 2.03 2.96 2.29 2.19	Ser71, (C-H-Bond) Lys234, (C-H-Bond)	3.79 3.53	12	14	−13.00
3	*Methicillin-resistant S. aureus*	Asn393, (H- Bond) Gln396, (H- Bond) Tyr499, (H- Bond) Gly282, (H- Bond) Leu252, (H- Bond) His251, (H- Bond) Tyr496, (H- Bond)	2.27 2.38 2.76 2.22 2.24 1.76 1.95	Lys281, (C-H bond) Gln396, (Metal-Acceptor) Tyr499,(Metal-Acceptor) Tyr496,(Pi-Donor Bond)	3.68 3.10 2.65 3.04	7	11	−12.60
4	*Methicillin-sensitive S. aureus*	Ser247, (H-Bond) Asp244, (H-Bond) Arg239, (H-Bond) Asp378, (H-Bond) Asp380, (H-Bond)	3.26 2.92 2.70 3.13 2.18	Gly240, (C-H bond) Asp244, (Metal-Acceptor) Asp378, (Metal-Acceptor)	2.91 2.84 3.15	5	8	−8.50

^*a*^ Ala: Alanine, Arg: Arginine, Asn: Asparagine Asp: Aspartic acid, Cys: Cysteine, Glu: Glutamic acid, Gln: Glutamine, Gly: Glycine, His: Histidine, Leu: Leucine, Lys: Lysine, Met: Methionine, Phe: Phenylalanine, Pro: Proline, Ser: Serine, Thr: Threonine, Tyr: Tyrosine, Val: Valine.

In addition, KPC-2 carbapenemase, a class A β-lactamase critically linked to carbapenem resistance in *K. pneumoniae*, serves as a key therapeutic target. Molecular docking analysis (Table 4) revealed robust-binding affinities of silver nanoparticles (AgCl-NPs: −13.00 kcal/mol; AgCr-NPs: −14.50 kcal/mol) to the KPC-2 active site. AgCl-NPs formed nine hydrogen bonds with residues Ser130, Asn214, Thr216, Arg220, Lys234, Thr235, Thr237, Glu276, Asp246, Val127, Gln128, and Asn245 (Fig. 11g through i), alongside two hydrophobic interactions (carbon-hydrogen bonds: Ser71, Lys234). In contrast, AgCr-NPs exhibited 13 hydrogen bonds, engaging residues Leu68, Cys69 (twice), Phe72, Arg161, Ser181, Ser182, Tyr264, Thr180, Phe66, Lys234, and Ala185 (Fig. 12g through i), as well as three hydrophobic interactions (carbon-hydrogen bond: Ser182; metal-acceptor bonds: Asp179, Asp157). Notably, shared catalytic residues Lys234, Thr235, Ser182, and Tyr247 played a pivotal role in stabilizing both nanoparticle interactions (Fig. 12g through i).

**TABLE 4:** Molecular interactions of AgCr-NPs with amino acids of the list of targets^*a*^

	Proteins	Hydrophilic interactions		Hydrophobic contacts		No. of H-Bonds	No. of total bonds	Affinity kcal mol−1
		Residue (H- Bond)	Length	Residue (bond type)	Length			
1	*A. baumannii*	Arg250, (H- Bond) Gln427, (H- Bond) Glu247, (H- Bond) Lys421, (H- Bond) Glu247, (H- Bond) Gln247, (H- Bond) Gln427, (H- Bond)	2.66 3.13 3.27 2.75 1.86 3.08 2.48	Ser251, (C-H-Bond) Ser251, (C-H-Bond) Glu247, (Metal-Acceptor)	2.85 3.19 2.82	7	10	−10.55
2	*K. pneumoniae*	Leu68, (H- Bond) Cys69, (H- Bond) Phe72, (H- Bond) Arg161, (H- Bond) Ser181, (H- Bond) Ser182, (H- Bond) Tyr264, (H- Bond) Cys69, (H- Bond) Thr180, (H- Bond) Phe66, (H- Bond) Lys234, (H- Bond) Ala185, (H- Bond)	2.40 3.01 2.79 2.19 2.41 2.50 1.85 3.37 2.63 3.08 2.79 2.07	Ser182, (C-H-Bond) Asp179, Metal-Acceptor) Asp157, Metal-Acceptor)	3.26 3.39 3.34	13	16	−14.50
3	*Methicillin-resistant Staphylococcus aureus*	Gly282, (H- Bond) Tyr366, (H- Bond) Asn393, (H- Bond) Tyr496, (H- Bond) His251, (H- Bond) Gly282, (H- Bond) Leu252, (H- Bond) Tyr499, (H- Bond) Pro497, (H- Bond)	2.37 2.23 2.50 2.72 2.82 3.09 3.07 2.24 2.74	Gly282, (C-H bond) Tyr499, (Metal-Acceptor) Tyr496,(Metal-Acceptor) Tyr496,(Pi-Donor Bond)	3.52 3.24 1.73 3.01	10	14	−12.80
4	*Methicillin-sensitive Staphylococcus aureus*	Asp378, (H-Bond) Asp236, (H-Bond) Asp380, (H-Bond) Ser274, (H-Bond) Asp244, (H-Bond)	3.07 3.36 2.85 2.82 1.68	Gly240, (C-H bond) Asp380, (C-H bond) Asp244, (Metal-Acceptor) Asp378, (Metal-Acceptor)	3.65 3.49 2.73 2.62	5	9	−9.50

^*a*^ Ala: Alanine, Arg: Arginine, Asn: Asparagine Asp: Aspartic acid, Cys: Cysteine, Glu: Glutamic acid, Gln: Glutamine, Gly: Glycine, His: Histidine, Leu: Leucine, Lys: Lysine, Phe: Phenylalanine, Pro: Proline, Ser: Serine, Thr: Threonine, Tyr: Tyrosine, Val: Valine.

Moreover, Penicillin-binding protein 2a (PBP2a), a central mediator of beta-lactam resistance in MRSA, enables pathogen survival by evading antibiotic targeting. Molecular docking analysis demonstrated strong binding affinities of silver nanoparticles to PBP2a, with AgCl-NPs at −12.60 kcal/mol and AgCr-NPs at −12.80 kcal/mol. AgCl-NPs formed seven hydrogen bonds with residues Asn393, Gln396, Tyr499, Gly282, Leu252, His251, and Tyr496 (Fig. 11g through i), alongside five hydrophobic interactions (carbon-hydrogen bonds: Lys281, Gln396; metal-acceptor bonds: Tyr499; pi-donor bond: Tyr496). In contrast, AgCr-NPs exhibited nine hydrogen bonds, engaging Gly282 (twice), Tyr366, Asn393, Tyr496, His251, Leu252, Tyr499, and Pro497, as well as seven hydrophobic interactions (carbon-hydrogen bonds: Gly282; metal-acceptor bonds: Tyr499, Tyr496; pi-donor bond: Tyr496). Key shared residues in the catalytic site Leu252, His251, Tyr366, and Tyr499 significantly stabilized both nanoparticle interactions (Fig. 12g through i).

In MSSA, Penicillin-Binding Protein 3 (PBP3) is vital for cell wall biosynthesis and a key target of β-lactam antibiotics. Its role in maintaining cell wall integrity and growth makes it an important focus for developing therapies against MSSA infections. Molecular docking analysis revealed that AgCl-NPs and AgCr-NPs exhibited binding affinities of −8.50 kcal/mol and −9.50 kcal/mol, respectively, toward PBP3. Specifically, AgCl-NPs formed five hydrogen bonds with residues Ser247, Asp244, Arg239, Asp378, and Asp380 (Fig. 11j through l), while AgCr-NPs also established five hydrogen bonds, interacting with Asp378, Asp236, Asp380, Ser274, and Asp244. In terms of hydrophobic interactions, AgCl-NPs displayed one carbon-hydrogen bond with Gly240 and two metal-acceptor bonds involving Asp244 and Asp378. In contrast, AgCr-NPs exhibited four hydrophobic contacts, including two carbon-hydrogen bonds with Gly240 and Asp380, as well as metal-acceptor bonds with Asp244 and Asp378 within the active site. Key residues in the catalytic site, Ser247, Asp244, Asp378, and Asp380, were identified as critical contributors to the enhanced binding affinity of both nanoparticles (Fig. 12j through l).

## DISCUSSION

The global rise of antibiotic resistance demands alternative antimicrobial strategies. This study synthesized Ag/AgCl and AgCr nanoparticles via PLE and evaluated their antimicrobial, anti-biofilm, and docking activities.

Characterization confirmed their structural morphology. XRD and Rietveld analysis revealed crystalline nanoscale structures of Ag/AgCl-NPs and AgCr-NPs. The XRD results of Ag/AgCl-NPs matched previous reports using *Azadirachta indica* (40). Furthermore, chromium incorporation led to AgCr-NPs with smaller crystallite sizes and distinct lattice parameters. SEM showed spherical particles (13–24 nm), and EDX confirmed elemental composition, including plant-derived C and O. Compared to Ag/AgCl-NPs, Cr doping resulted in a reduced silver percentage, suggesting possible surface modification by phytochemicals or structural incorporation of chromium. Similar findings were reported for *Pelargonium sidoides*-derived Ag-NPs (23). Additionally, O, Cl, and C binding energy peaks likely arise from plant organics or drying impurities (23, 24). XPS analysis confirmed the presence of multiple chemical elements, with Cl peaks reflected biomolecule interactions with Ag-NPs or plant-derived Cl− from photosynthesis and homeostasis (41, 42). XPS and FTIR validated the oxidation states of Ag and Cr and revealed Ag–O and C–O bonds, indicating the phytomolecules role in NP surface chemistry.

The optical properties of the NPs revealed strong SPR at 428–430 nm and reduced band gap energies, particularly in AgCr-NPs (1.70 eV). SPR peaks between 410 and 450 nm are characteristic of Ag-NPs and linked to spherical shapes (43). According to Lopez-Ayuso et al., absorption bands range between 407 and 431 nm, confirming the reduction of Ag^+^ to Ag^0^ (44). An SPR at 421 nm was also noted for chromium oxide NPs, while minor peaks may reflect impurities or transitions (45). Band gap reduction was similarly observed in Ag-ZnO nanoparticles, where band gaps decreased from 3.37 eV to 2.40 eV with increasing silver content in the ZnO nanoparticles (46). Photoluminescence studies revealed oxygen vacancies and Ag–Ag interactions, which contribute to reactive oxygen species (ROS) generation, a key mechanism in nanoparticle antibacterial activity (47–49). Phytochemical analysis of PLE identified flavonoids, phenols, terpenoids, and steroids, which acted as reducing and stabilizing agents during nanoparticle synthesis (50). These metabolites supported the green formation of Ag/AgCl and AgCr NPs and likely enhanced their antibacterial and anti-biofilm activities. Similar findings from *P. graveolens* aqueous extract in India reported flavonoids, phenols, tannins, saponins, steroids, glycosides, terpenoids, and reducing sugars, but in the absence of tannins, phlorotannins, anthraquinones, and starch (51).

Both Ag/AgCl-NPs and AgCr-NPs exhibit strong antibacterial activity. Extracts from *P. betulinum* and *P. capitatum* showed activity against *K. pneumoniae* with an MIC of 1,000 µg/mL (17). The MIC results obtained were in agreement to those reported in the literature where Ag-NPs synthesized from various plant sources showed MICs ranging from 125 to 250 µg/mL against bacteria like *S. aureus*, MRSA, and *K. pneumoniae* (52–54). The findings also align with previous results, where Ag-NPs showed an MIC of 125 µg/mL against a clinical isolate of *A. baumannii* (55). The antimicrobial effect of chromium III oxide (Cr_2_O_3_) nanoparticles, synthesized via sol-gel method, was evaluated against *K. pneumoniae*, revealing an MIC of 2.5 mg/mL (56). The bactericidal capabilities of NPs are determined by the source of NPs, particle synthesis method, particle size, shape, charge, dose, and exposure time of the bacteria (57). Time-kill studies revealed that AgCr-NPs act rapidly within the first hour, while Ag/AgCl-NPs have a slower but sustained effect, likely due to differences in their antimicrobial mechanisms. AgCr-NPs and PLE may quickly disrupt bacterial membranes or metabolic pathways, whereas Ag/AgCl-NPs cause a prolonged lag phase through gradual ion release and reactive oxygen species production (58). This aligns with previous reports that describe Ag-NPs delaying the transition from lag to exponential phase**,** thereby exerting a bacteriostatic effect (51). Additionally, Ag/AgCl-NPs are more effective against Gram-positive bacteria (MSSA and MRSA), while AgCr-NPs show enhanced activity against Gram-negative bacteria (*K. pneumoniae* and *A. baumannii*), possibly due to differences in bacterial cell wall structures and susceptibility to oxidative stress (59). Importantly, both nanoparticles disrupted biofilm formation in a concentration-dependent manner, outperforming doxycycline in several cases. PLE also showed strong anti-biofilm activity, likely due to phytochemicals that interfere with quorum sensing, EPS production, and bacterial adhesion as previously reported (60). In addition, Ag/AgCl-NPs showed higher anti-biofilm activity at 24 h, especially against MRSA and *K. pneumoniae*, likely due to rapid silver ion release that penetrates biofilms through aqueous channels, allowing them to reach deeper bacterial layers that are typically inaccessible to conventional antibiotics. In contrast, AgCr-NPs were more effective against MRSA at 48 h, suggesting a sustained release or improved interaction with mature biofilms, possibly due to surface charge changes from chromium doping (61). PLE, Ag/AgCl-NPs, and AgCr-NPs exhibited better biofilm inhibition compared to doxycycline against MSSA and *K. pneumoniae* at both 24 and 48 h. Additionally, AgCr-NPs proved to be more effective than doxycycline against MRSA and *A. baumannii*. The results suggest that nanoparticles interfere with multiple stages of biofilm development such as adhesion, colonization, and maturation, through mechanisms such as membrane disruption, ROS generation, ion release, and quorum sensing inhibition (62). This dual action, combining fast-acting membrane effects with prolonged intracellular toxicity from metal ions (Ag^+^, Cr³^+^), highlights their potential as effective anti-biofilm agents. Molecular docking analysis revealed that Ag/AgCl and AgCr nanoparticles strongly bind to key bacterial enzymes like PBPs and β-lactamases, potentially disrupting cell wall synthesis and overcoming resistance to β-lactam antibiotics. Similarly, high binding scores against KPC-2 carbapenemase in *K. pneumoniae* highlight their potential to inhibit carbapenem hydrolysis, one of the most critical resistance mechanisms in Gram-negative pathogens. These findings are consistent with recent computational studies demonstrating the potential of silver-based nanostructures to target bacterial enzymes. Darwich et al. (61) used molecular docking to analyze interactions with penicillin-binding proteins (PBPs), while El-Sayed et al. (61) confirmed that silver-based nanostructures effectively disrupt *A. baumannii* PBPs, demonstrating their strong antibacterial potential (61). In addition, Darwich et al. (61) demonstrated that Ag-NPs can inhibit KPC-2 carbapenemase, while Ikram et al. (63) highlighted the efficacy of nanomaterials in targeting cell wall synthesis enzymes (61). Similarly, Darwich et al. (61) confirmed that Ag-NPs disrupt penicillin-binding proteins in *Enterococcus faecalis*, highlighting the wide antibacterial potential of nanoparticle-based treatments (61). These results corroborate computational studies by Darwich et al. (61) showing that Ag-NPs strongly inhibit *S. aureus* PBPs, demonstrating their ability to overcome traditional antibiotic resistance (61). These findings are consistent with the work of Abdel-Wahed et al. (64), who utilized in-silico simulations to show that Ag-NPs inhibit multidrug-resistant bacteria, supporting the antibacterial potential found in this study’s docking analysis (61). Overall, this study shows that Ag/AgCl and AgCr NPs possess favorable structural and biological properties, including broad-spectrum antibacterial and anti-biofilm activity.

### Conclusion

This study demonstrates an eco-friendly synthesis of Ag/AgCl-NPs and AgCr-NPs using *Pelargonium graveolens* leaf extract, producing spherical nanoparticles with notable antimicrobial activity against various Gram-positive and Gram-negative bacteria. Molecular docking suggests these nanoparticles effectively bind to key bacterial proteins, potentially disrupting cell wall synthesis and offering a promising strategy against drug-resistant pathogens. However, limitations, such as unknown cytotoxicity, and challenges in large-scale production highlight the need for further research before clinical or commercial application.

## MATERIALS AND METHODS

### Preparation of *Pelargonium graveolens* leaf extracts

*P. graveolens* was obtained from Barja, Iklim Al Kharoub, Mount Lebanon (33.65000°N, 35.43167°E). *P. graveolens’* fresh leaves were vigorously washed with distilled water to remove soil and dust. The leaves were allowed to shade-dry for 30 min at 25°C before being cut into small fragments. Then, 2% of PLE was prepared using distilled water and then agitated in an incubator shaker at 120 rpm for 30 min at 60°C (61). After cooling, the extract was filtered using a vacuum pump, and the resulting extract was stored in dark bottles.

### Qualitative phytochemical analysis

Qualitative phytochemical screening of PLE was conducted following Sawant et al. (65) for anthraquinones, phenols, flavonoids, saponins, quinones, terpenoids, and steroids. Anthraquinones were tested by mixing 1 mL of PLE with 1% H_2_SO_4_, extracting with chloroform/benzene, and adding diluted ammonia, where a pink color indicated positivity. Phenols were identified by mixing the extract with distilled water and 10% ferric chloride, producing blue/green coloration. Flavonoids were detected by adding 2% NaOH to PLE, yielding a yellow color that disappeared upon addition of diluted HCl. Saponins were confirmed by mixing PLE with water and observing persistent foam after 15 min. Terpenoids were tested by treating PLE with chloroform and concentrated H_2_SO_4_, where a red-brown interface appeared. Steroids were identified using acetic anhydride and concentrated H_2_SO_4_, producing a reddish-brown ring and green interface.

### Synthesis of Ag/AgCl and AgCr-NPs

0.042 g of 1 mM silver nitrate (AgNO_3_) and 0.1 g of 1 mM chromium (III) nitrate nonahydrate (Cr(NO_3_)_3_·9H_2_O) were added to separate flasks, each containing 250 mL of *P. graveolens* leaf extract. The flasks with the mixtures were subjected to heating at 60°C for 30 min with continuous stirring. Subsequently, the solutions were incubated in the dark at room temperature for 90 min (66). The resulting Ag/AgCl and Cr nanoparticles were obtained through centrifugation at 10,000 rpm for 30 min. The synthesized Ag/AgCl-NPs were then dried at 40°C for 24 h. In addition, to produce AgCr-NPs, the previously mentioned nanoparticles were mixed and heated at 60°C for 30 min with continuous stirring, followed by centrifugation at 10,000 rpm for 30 min, and the final product was dried at 40°C for 24 h.

### Characterization of nanoparticles

The crystalline phase was determined using X-ray diffraction (XRD) PANALYTICAL CO’s Xpert Pro software (Panalytical business, Holland) with a Cu-*K*_α_ target. The XRD pattern was recorded at 1.5406 Å wavelength, 40 mA current, and 45 kV voltage. The XRD data were recorded over a 2*θ* range from 20° to 80°. The XRD profiles were optimized using the MAUD program. The elemental composition and surface morphology were evaluated using scanning electron microscopy (SEM) with energy dispersive X-ray spectroscopy (EDX). These measurements were carried out at 10 keV in different sample regions with a JEOL JCM-6000PLUS fitted with an EX-54450U1S61 detector. K-Alpha used monochromatic X-ray photons to evaluate the X-ray photo-induced spectrometer (XPS) (Thermo Fisher Scientific, USA). The energy of the former radiation ranged from −10 to 1,350 eV, whereas the pass energy was 200 and 50 eV for the whole survey and high-resolution spectra, respectively. The Nicolet i*S*5 Fourier transform infrared spectroscopy (FTIR) was used to determine the functional groups in the range of 4,000–500 cm^−1^. Ultraviolet-visible spectroscopy (UV-Vis) V-670 spectrophotometer (Jasco, Japan) was utilized to determine the absorption wavelength of Ag/AgCl-NPs and AgCr-NPs. The photoluminescence spectroscopy (PL) was studied by a Jasco FP-8600 spectrofluorometer (Jasco, Japan) equipped with a xenon laser (Xe) at an excitation wavelength of 410 nm for Ag/AgCl-NPs and AgCr-NPs.

### Determination of the minimum inhibitory concentration (MIC) and the minimum bactericidal concentration

The antibacterial activity of Ag/AgCl-NPs and AgCr-NPs against four bacterial isolates*, Methicillin-susceptible Staphylococcus aureus* (MSSA), *Methicillin-resistant Staphylococcus aureus* (MRSA)*, Klebsiella pneumoniae* (*K. pneumoniae*), and *Acinetobacter baumannii* (*A. baumannii*) was assessed using the broth dilution method. Bacterial strains were obtained from the Microbiology Department at BAU. MIC values were determined through twofold serial dilutions of nanoparticles ranging from 0.488 to 1,000 µg/mL, using bacterial suspensions standardized to 0.5 McFarland. In a 96-well plate, 100 µL of nanoparticles, 90 µL nutrient broth, and 10 µL bacterial suspension were combined for a total of 200 µL and incubated at 37°C for 24 h. The optical density (OD) was recorded at 595 nm by a MultiskanTM FC-ELISA microplate reader (ThermoFisher Scientific, USA) (67). The MIC was known as the smallest concentration showing no visible microbial growth (68). Experiments were performed in triplicate, with mean values recorded. To determine the MBC, 10 µL from each well was plated into Muller Hinton agar and incubated for 24 h at 37°C.

### Time kill test

Time-kill studies were conducted to assess the duration required for the tested extract and nanoparticles to inhibit bacterial growth (69). In sterile 96-well plates, 90 µL nutrient broth was mixed with 10 µL bacterial suspensions (0.5 McFarland), and then 100 µL of the nanoparticles MIC was added (70). Plates were incubated at 37°C, and bacterial growth was monitored by measuring the optical density (OD) at 595  nm using Multiskan FC-ELISA microplate reader at 0, 1, 2, 4, and 24 h. Experiments were done in triplicate.

### Antibiofilm screening

#### Inhibition of biofilm formation—prevention of initial bacterial cell attachment

The biofilm inhibition assay has been conducted to evaluate the efficacy of PLE and NPs in preventing the initial adhesion of cells (71). A 100 µL aliquot of cultures containing 1.0  ×  10^6^  CFU/mL of MSSA, MRSA, *K. pneumoniae*, and *A. baumannii* was introduced to 96-well plates and incubated at 37°C for 4 h. Subsequently, 100 µL aliquots of PLE and NPs with a concentration ranging from 0.488 to 1,000 μg/mL were added to the 96-well plates, which had subsequently incubated at 37°C for 24 and 48 h. Doxycycline served as a positive control (72). Biofilm biomass was measured using crystal violet staining: after incubation, wells were washed and dried for 45 min at 60°C, and 100 µL of 1% crystal violet was added. Then, the wells were incubated for 15 min at room temperature before being washed to remove excess dye. At this point, biofilms emerged as purple rings on the well walls. To de-stain the wells, a 100 µL aliquot of 95% ethanol was used. The absorbance was measured at 595 nm using a Multiskan FC-ELISA microplate reader (ThermoFisher Scientific, USA). The percentage of biofilm (%) was calculated using the mentioned formula (1) (71):


(1) $$\%\text{ Inhibition }=\frac{OD\;(\text{Negative control})-OD\;(\text{Experimental})}{OD\;(\text{Negative control})}\times 100$$

#### Inhibition of the growth of pre-formed biofilms—assessment of biofilm eradication

The extract and the synthesized NPs capacity to prevent additional biofilm growth or destroy existing biofilms were studied. A 100 μL of standardized cultures (1.0 × 10^6^ CFU/mL) of MSSA, MRSA, *K. pneumoniae*, and *A. baumannii* was introduced to 96-well plates and incubated at 37 °C for 24 h and 48 h. After the incubation period, 100 μL aliquots of PLE or NPs were added, and the plates were incubated at 37°C for 24 h. Doxycycline served as a positive control (72). The biofilm biomass was assessed using the modified crystal violet (CV) staining technique, and the percentage of eradication was calculated using the following formula (2) (73).


(2) $$\%\text{ Eradication }=\frac{OD\;(\text{Negative control})-OD\;(\text{Experimental})}{OD\;(\text{Negative control})}\times 100$$

### Molecular docking simulation

Protein receptors exhibiting antimicrobial properties were retrieved from the RCSB Protein Data Bank, as outlined in Table S5. The crystal structures of receptors were prepared by removing water molecules, ions, and pre-existing ligands using PyMOL software. Subsequently, hydrogen atoms were added to the structures with MG Tools to ensure proper protonation states. This standardized preparation was uniformly applied across all protein structures, which were then saved in the PDBQT format. The structures of (AgCl-NPs) and (AgCr-NPs) were initially constructed in the 2D Mol2 format and subsequently converted into the 3D PDB format using Open Babel. Ligand-centered grid maps, with dimensions of 90 Å × 90 Å × 90 Å, were generated using the AutoGrid program to define the potential protein-ligand interaction regions, with all other parameters maintained at their default settings. The 2D interactions, including hydrogen bonds, hydrophobic contacts, and bond distances for each docking pose, were analyzed, and the results were visualized graphically using BIOVIA Discovery Studio (version 4.5).

### Statistical analysis

The statistical analyses were carried out using the Excel software (version: 14.0.4760.1000, 32-bit, 2010). The results are expressed as the mean of three setups ± standard error of the mean (SEM) (refer to supplementary information). Each experiment was repeated at least three times, and the data were analyzed using a *t*-test, with a *P*-value of less than 0.05 considered significant. The graphical representations were created using the Origin software (64-bit edition, 2022).

## SUPPLEMENTAL MATERIAL

Tables S1 to S5 spectrum.01142-25-s0001.pdf MIC results, Estimated p-value, and significance levels of the different nanoparticles at different time-points. List of targets, PDB IDs, resolution, and active site coordinates.

ASM does not own the copyrights to Supplemental Material that may be linked to, or accessed through, an article. The authors have granted ASM a non-exclusive, world-wide license to publish the Supplemental Material files. Please contact the corresponding author directly for reuse.
