# Supplementary material for: Unveiling the antibacterial potential of green-synthesized silver chloride and silver chromium nanoparticles from Pelargonium graveolens extract through molecular docking and bioactivity studies
Source: Microbiol Spectr. 2025 Nov 25;14(1):e01142-25. doi: 10.1128/spectrum.01142-25 (PMC12772281; doi:10.1128/spectrum.01142-25)
Supplement: Tables S1 to S5 — MIC results, Estimated p-value, and significance levels of the different nanoparticles at different time-points. List of targets, PDB IDs, resolution, and active site coordinates. [file spectrum.01142-25-s0001.pdf]

Supplementary Data

Table S1. The MIC results (*p*-value and significance levels) of PLE, Ag/AgCl-NPs, and AgCr-NPs

**Samples**	**Bacterial isolates**			
	**MSSA**	**MRSA**	***K. pneumoniae***	***A. baumannii***
PLE	0.009**	0.005**	0.003**	0.002**
Ag/AgCl-NPs	0.003**	0.00003***	0.005**	0.001***
AgCr-NPs	0.002**	0.0005***	0.006**	0.0007***

***p*-values less than 0.05 are statistically significant.** $^{*}p < 0.05$, $^{**}p < 0.01$, $^{***}p < 0.001$.

Table S2. Estimated *p*-value and significance levels of the time kill findings of the PLE, Ag/AgCl-NPs, and AgCr-NPs.

**Samples**		**Bacterial isolates**			
		**MSSA**	**MRSA**	***K. pneumoniae***	***A. baumannii***
		**Mean±SEM**	**Mean±SEM**	**Mean±SEM**	**Mean±SEM**
PLE	T_0	0.002**	0.007**	0.002**	0.003**
	T_1	0.004**	0.005**	0.003**	0.005**
	T_2	0.003**	0.003**	0.007**	0.001**
	T_4	0.0008***	0.0008***	0.002**	0.002**
	T_{24}	0.003**	0.004**	0.005**	0.002**
Ag/AgCl-NPs	T_0	0.001**	0.005**	0.002**	0.0005***
	T_1	0.001**	0.004**	0.002**	0.001**
	T_2	0.001**	0.002**	0.0005***	0.0008***
	T_4	0.007**	0.001**	0.001**	0.001**
	T_{24}	0.006**	0.003**	0.009**	0.003**
AgCr-NPs	T_0	0.002**	0.001**	0.001**	0.002**
	T_1	0.001**	0.0006***	0.002**	0.002**
	T_2	0.0005***	0.0008***	0.0008***	0.003**
	T_4	0.001**	0.0005***	0.001**	0.002**
	T_{24}	0.002**	0.001**	0.002**	0.001**

***p*-values less than 0.05 are statistically significant.** $^{*}p < 0.05$, $^{**}p < 0.01$, $^{***}p < 0.001$.

Table S3(a). Estimated *p*-value and significance levels of Ag/AgCl-NPs against biofilm formation by gram-negative and gram-positive bacteria at 24 h.

**Concentration** (µg/mL)	**Ag/AgCl-NPs**			
	**MSSA**	**MRSA**	***K. pneumoniae***	***A. baumannii***
**10000**	0.001**	0.006**	0.002**	0.007**
**500**	0.002**	0.010*	0.002**	0.006**
**250**	0.005**	0.008**	0.020*	0.012*
**125**	0.004**	0.010*	0.020*	0.015*
**62.5**	0.012*	0.012*	0.024*	0.026*
**31.25**	0.018*	0.009**	0.025*	0.030*
**15.62**	0.014*	0.019*	0.024*	0.041*
**7.8125**	0.024*	0.020*	0.047*	0.043*
**3.9**	0.030*	0.018*	0.009**	0.039*
**1.95**	0.033*	0.014*	0.013*	0.046*
**0.97**	0.023*	0.013*	0.023*	0.027*
**0.488**	0.030*	0.004**	0.0004***	0.005**

***p*-values less than 0.05 are statistically significant. $^{*}p < 0.05$, $^{**}p < 0.01$, $^{***}p < 0.001$, ns: not significant.**

Table S3(b). Estimated *p*-value and significance levels of AgCr-NPs against biofilm formation by gram-negative and gram-positive bacteria at 24 h.

**Concentration** (µg/mL)	**AgCr-NPs**			
	**MSSA**	**MRSA**	***K. pneumoniae***	***A. baumannii***
**10000**	0.022*	0.016*	0.001**	0.027*
**500**	0.027*	0.018*	0.005**	0.028*
**250**	0.024*	0.017*	0.004**	0.032*
**125**	0.026*	0.023*	0.004**	0.042*
**62.5**	0.027*	0.028*	0.006**	0.035*
**31.25**	0.029*	0.023*	0.004**	0.028*
**15.62**	0.035*	0.026*	0.0003***	0.042*
**7.8125**	0.037*	0.030*	0.0003***	0.047*
**3.9**	0.038*	0.031*	0.0003***	0.046*
**1.95**	0.045*	0.046*	0.020**	0.047*
**0.97**	0.031*	0.025*	0.012*	0.047*
**0.488**	0.030*	0.039*	0.045*	0.038*

***p*-values less than 0.05 are statistically significant. $^{*}p < 0.05$, $^{**}p < 0.01$, $^{***}p < 0.001$, ns: not significant.**

Table S3(c). Estimated *p*-value and significance levels of PLE against biofilm formation by gram-negative and gram-positive bacteria at 24 h.

**Concentration** (µg/mL)	**PLE**			
	**MSSA**	**MRSA**	***K. pneumoniae***	***A. baumannii***
**10000**	0.026*	0.019*	0.010*	0.007**
**500**	0.024*	0.015*	0.011*	0.006**
**250**	0.033*	0.017*	0.009**	0.005**
**125**	0.037*	0.024*	0.008**	0.020*
**62.5**	0.041*	0.027*	0.009**	0.018*
**31.25**	0.047*	0.030*	0.008**	0.009**
**15.62**	0.044*	0.040*	0.0006***	0.005**
**7.8125**	0.047*	0.024*	0.0008***	0.008**
**3.9**	0.044*	0.036*	0.0007***	0.009**
**1.95**	0.047*	0.034*	0.006**	0.034*
**0.97**	0.048*	0.042*	0.0001***	0.044*
**0.488**	0.034*	0.030*	0.013*	0.020*

***p*-values less than 0.05 are statistically significant.** $^{*}p < 0.05$, $^{**}p < 0.01$, $^{***}p < 0.001$, **ns: not significant.**

Table S3(d). Estimated *p*-value and significance levels of doxycyline against biofilm formation by gram-negative and gram-positive bacteria at 24 h.

**Concentration** (µg/mL)	**Doxycyline**			
	**MSSA**	**MRSA**	***K. pneumoniae***	***A. baumannii***
**10000**	0.001**	0.008**	0.001**	0.006**
**500**	0.002**	0.009**	0.001**	0.007**
**250**	0.006**	0.002**	0.002**	0.008**
**125**	0.006**	0.003**	0.002**	0.010*
**62.5**	0.017*	0.003**	0.001**	0.008**
**31.25**	0.008**	0.001**	0.002**	0.013*
**15.62**	0.007**	0.002**	0.002**	0.024*
**7.8125**	0.021*	0.002**	0.004**	0.028*
**3.9**	0.009**	0.003**	0.019*	0.034*
**1.95**	0.113	0.039*	0.006**	0.030*
**0.97**	0.164	0.033*	0.001**	0.035*
**0.488**	0.027*	0.004**	0.0002***	0.028*

***p*-values less than 0.05 are statistically significant.** $^{*}p < 0.05$, $^{**}p < 0.01$, $^{***}p < 0.001$, **ns: not significant.**

Table S3(e). Estimated *p*-value and significance levels of Ag/AgCl-NPs against biofilm formation by gram-negative and gram-positive bacteria at 48 h.

**Concentration** (µg/mL)	**Ag/AgCl-NPs**			
	**MSSA**	**MRSA**	***K. pneumoniae***	***A. baumannii***
**10000**	0.046*	0.028*	0.009**	0.004**
**500**	0.043*	0.019*	0.014*	0.011*
**250**	0.044*	0.029*	0.018*	0.005**
**125**	0.047*	0.044*	0.018*	0.036*
**62.5**	0.048*	0.032*	0.022*	0.045*
**31.25**	0.046*	0.040*	0.026*	0.048*
**15.62**	0.048*	0.041*	0.036*	0.045*
**7.8125**	0.047*	0.045*	0.039*	0.039*
**3.9**	0.047*	0.043*	0.039*	0.047*
**1.95**	0.046*	0.040*	0.048*	0.047*
**0.97**	0.042*	0.040*	0.033*	0.034*
**0.488**	0.037*	0.047*	0.037*	0.003**

***p*-values less than 0.05 are statistically significant. $^{*}p < 0.05$, $^{**}p < 0.01$, $^{***}p < 0.001$, ns: not significant.**

Table S3(f). Estimated *p*-value and significance levels of AgCr-NPs against biofilm formation by gram-negative and gram-positive bacteria at 48 h.

**Concentration** (μg/mL)	**AgCr-NPs**			
	**MSSA**	**MRSA**	***K. pneumoniae***	***A. baumannii***
**10000**	0.005**	0.003**	0.048*	0.003**
**500**	0.009**	0.003**	0.048*	0.004**
**250**	0.013*	0.003**	0.046*	0.013*
**125**	0.013*	0.003**	0.047*	0.014*
**62.5**	0.020*	0.003**	0.046*	0.017*
**31.25**	0.023*	0.007**	0.045*	0.001**
**15.62**	0.018*	0.007**	0.046*	0.014*
**7.8125**	0.020*	0.008**	0.048*	0.032*
**3.9**	0.007**	0.008**	0.048*	0.038*
**1.95**	0.031*	0.014*	0.041*	0.037*
**0.97**	0.032*	0.021*	0.046*	0.038*
**0.488**	0.043*	0.049*	0.048*	0.043*

***p*-values less than 0.05 are statistically significant. $^{*}p < 0.05$, $^{**}p < 0.01$, $^{***}p < 0.001$, ns: not significant.**

Table S3(g). Estimated *p*-value and significance levels of PLE against biofilm formation by gram-negative and gram-positive bacteria at 48 h.

**Concentration** (µg/mL)	**PLE**			
	**MSSA**	**MRSA**	***K. pneumoniae***	***A. baumannii***
**10000**	0.005**	0.0009***	0.0005***	0.0006***
**500**	0.007**	0.0006***	0.0001***	0.002**
**250**	0.007**	0.001**	0.0008***	0.002**
**125**	0.010*	0.0007***	0.0001***	0.002**
**62.5**	0.044*	0.0006***	0.0001***	0.048*
**31.25**	0.044*	0.0003***	0.0001***	0.048*
**15.62**	0.049*	0.003**	0.0008***	0.046*
**7.8125**	0.048*	0.004**	0.001**	0.045*
**3.9**	0.044*	0.005**	0.001**	0.045*
**1.95**	0.047*	0.004**	0.001**	0.045*
**0.97**	0.049*	0.042*	0.0008***	0.047*
**0.488**	0.039*	0.042*	0.0008***	0.043*

***p*-values less than 0.05 are statistically significant. $^{*}p < 0.05$, $^{**}p < 0.01$, $^{***}p < 0.001$, ns: not significant.**

Table S3(h). Estimated *p*-value and significance levels of doxycyline against biofilm formation by gram-negative and gram-positive bacteria at 48 h.

**Concentration** (µg/mL)	**Doxycyline**			
	**MSSA**	**MRSA**	***K. pneumoniae***	***A. baumannii***
**10000**	0.038*	0.019*	0.006*	0.004**
**500**	0.039*	0.001**	0.031*	0.006**
**250**	0.047*	0.001**	0.036*	0.006**
**125**	0.048*	0.023*	0.032*	0.014*
**62.5**	0.044*	0.034*	0.030*	0.019*
**31.25**	0.035*	0.023*	0.037*	0.019*
**15.62**	0.044*	0.048*	0.044*	0.029*
**7.8125**	0.040*	0.029*	0.042*	0.047*
**3.9**	0.048*	0.019*	0.039*	0.039*
**1.95**	0.039*	0.032*	0.024*	0.037*
**0.97**	0.036*	0.042*	0.016*	0.026*
**0.488**	0.048*	0.010*	0.004**	0.044*

***p*-values less than 0.05 are statistically significant.** $^{*}p < 0.05$, $^{**}p < 0.01$, $^{***}p < 0.001$, **ns: not significant.**

Table S4(a). Estimated *p*-value and significance levels of Ag/AgCl-NPs against biofilm destruction by gram-negative and gram-positive bacteria at 24 h.

**Concentration** (μg/mL)	**Ag/AgCl-NPs**			
	**MSSA**	**MRSA**	***K. pneumoniae***	***A. baumannii***
**10000**	0.0002***	0.00009***	0.0008***	0.029*
**500**	0.0004***	0.00008***	0.0002***	0.017*
**250**	0.001**	0.00001***	0.002**	0.055*
**125**	0.002**	0.00003***	0.004**	0.023*
**62.5**	0.004**	0.00002***	0.003**	0.041*
**31.25**	0.008**	0.0002***	0.002**	0.034*
**15.62**	0.005**	0.016*	0.006**	0.005**
**7.8125**	0.016*	0.023*	0.009**	0.023*
**3.9**	0.001**	0.045*	0.010*	0.006**
**1.95**	0.0005***	0.043*	0.011*	0.004**
**0.97**	0.023*	0.044*	0.010*	0.003**
**0.488**	0.004**	0.0009***	0.029*	0.003**

***p*-values less than 0.05 are statistically significant. $^{*}p < 0.05$, $^{**}p < 0.01$, $^{***}p < 0.001$, ns: not significant.**

Table S4(b). Estimated *p*-value and significance levels of AgCr-NPs against biofilm destruction by gram-negative and gram-positive bacteria at 24 h.

**Concentration** (µg/mL)	**AgCr-NPs**			
	**MSSA**	**MRSA**	***K. pneumoniae***	***A. baumannii***
**10000**	0.004**	0.024*	0.0001***	0.035*
**500**	0.006**	0.040*	0.004**	0.022*
**250**	0.009**	0.038*	0.001**	0.036*
**125**	0.012*	0.035*	0.001**	0.037*
**62.5**	0.011*	0.032*	0.007**	0.028*
**31.25**	0.012*	0.035*	0.005**	0.028*
**15.62**	0.009**	0.027*	0.004**	0.025*
**7.8125**	0.022*	0.043*	0.039*	0.035*
**3.9**	0.013*	0.042*	0.023*	0.040*
**1.95**	0.016*	0.027*	0.011*	0.045*
**0.97**	0.033*	0.002**	0.037*	0.019*
**0.488**	0.029*	0.020*	0.017*	0.036*

***p*-values less than 0.05 are statistically significant.** $^{*}p < 0.05$, $^{**}p < 0.01$, $^{***}p < 0.001$, **ns: not significant.**

Table S4(c). Estimated *p*-value and significance levels of PLE against biofilm destruction by gram-negative and gram-positive bacteria at 24 h.

**Concentration** (µg/mL)	**PLE**			
	**MSSA**	**MRSA**	***K. pneumoniae***	***A. baumannii***
**10000**	0.001**	0.022**	0.008**	0.044*
**500**	0.001**	0.026**	0.008**	0.048*
**250**	0.001**	0.020**	0.011*	0.048*
**125**	0.0001****	0.023**	0.024*	0.046*
**62.5**	0.001**	0.029**	0.027*	0.046*
**31.25**	0.003**	0.042**	0.041*	0.047*
**15.62**	0.002**	0.048**	0.016*	0.048*
**7.8125**	0.021*	0.039**	0.025*	0.048*
**3.9**	0.033*	0.037**	0.029*	0.046*
**1.95**	0.028*	0.041**	0.041*	0.043*
**0.97**	0.037*	0.043**	0.044*	0.047*
**0.488**	0.016*	0.045**	0.034*	0.047*

***p*-values less than 0.05 are statistically significant. $^{*}p < 0.05$, $^{**}p < 0.01$, $^{***}p < 0.001$, ns: not significant.**

Table S4(d). Estimated *p*-value and significance levels of doxycyline against biofilm destruction by gram-negative and gram-positive bacteria at 24 h.

**Concentration** (µg/mL)	**Doxycyline**			
	**MSSA**	**MRSA**	***K. pneumoniae***	***A. baumannii***
**10000**	0.004**	0.002**	0.0001***	0.007**
**500**	0.004**	0.006**	0.0008***	0.008**
**250**	0.005**	0.007**	0.0008***	0.007**
**125**	0.012*	0.002**	0.003**	0.006**
**62.5**	0.025*	0.003**	0.004**	0.008**
**31.25**	0.020*	0.003**	0.004**	0.008**
**15.62**	0.018*	0.010*	0.014*	0.007**
**7.8125**	0.021*	0.002**	0.040*	0.006**
**3.9**	0.038*	0.003**	0.043*	0.005**
**1.95**	0.043*	0.007**	0.020*	0.041*
**0.97**	0.042*	0.042*	0.040*	0.007**
**0.488**	0.048*	0.041*	0.033*	0.007**

***p*-values less than 0.05 are statistically significant. $^{*}p < 0.05$, $^{**}p < 0.01$, $^{***}p < 0.001$, ns: not significant.**

Table S4(e). Estimated *p*-value and significance levels of Ag/AgCl-NPs against biofilm destruction by gram-negative and gram-positive bacteria at 48 h.

**Concentration** (μg/mL)	**Ag/AgCl-NPs**			
	**MSSA**	**MRSA**	***K. pneumoniae***	***A. baumannii***
**10000**	0.004**	0.001**	0.003**	0.008**
**500**	0.010*	0.003**	0.003**	0.007**
**250**	0.007**	0.001**	0.009**	0.022*
**125**	0.008**	0.0008***	0.020*	0.027*
**62.5**	0.019*	0.0007***	0.020*	0.025*
**31.25**	0.014*	0.001**	0.008**	0.029*
**15.62**	0.026*	0.003**	0.028*	0.027*
**7.8125**	0.029*	0.005**	0.042*	0.034*
**3.9**	0.031*	0.007**	0.040*	0.045*
**1.95**	0.043*	0.006**	0.044*	0.046*
**0.97**	0.043*	0.007**	0.042*	0.042*
**0.488**	0.047*	0.048*	0.044*	0.042*

***p*-values less than 0.05 are statistically significant. $^{*}p < 0.05$, $^{**}p < 0.01$, $^{***}p < 0.001$, ns: not significant.**

Table S4(f). Estimated *p*-value and significance levels of AgCr-NPs against biofilm destruction by gram-negative and gram-positive bacteria at 48 h.

**Concentration** (µg/mL)	**AgCr-NPs**			
	**MSSA**	**MRSA**	***K. pneumoniae***	***A. baumannii***
**10000**	0.014*	0.008**	0.0002***	0.002**
**500**	0.015*	0.010*	0.0004***	0.013*
**250**	0.016*	0.011*	0.0007***	0.015*
**125**	0.024*	0.013*	0.0008***	0.039*
**62.5**	0.031*	0.010*	0.001**	0.036*
**31.25**	0.041*	0.016*	0.013*	0.039*
**15.62**	0.040*	0.016*	0.039*	0.043*
**7.8125**	0.038*	0.042*	0.047*	0.044*
**3.9**	0.041*	0.045*	0.044*	0.048*
**1.95**	0.030*	0.048*	0.042*	0.047*
**0.97**	0.034*	0.048*	0.047*	0.048*
**0.488**	0.047*	0.044*	0.040*	0.044*

***p*-values less than 0.05 are statistically significant. $^{*}p < 0.05$, $^{**}p < 0.01$, $^{***}p < 0.001$, ns: not significant.**

Table S4(g). Estimated *p*-value and significance levels of PLE, and doxycyline against biofilm destruction by gram-negative and gram-positive bacteria at 48 h.

**Concentration** (µg/mL)	**PLE**			
	**MSSA**	**MRSA**	***K. pneumoniae***	***A. baumannii***
**10000**	0.001**	0.022*	0.008**	0.044*
**500**	0.001**	0.026*	0.008**	0.048*
**250**	0.001**	0.020*	0.011*	0.048*
**125**	0.0001***	0.023*	0.024*	0.046*
**62.5**	0.001**	0.029*	0.027*	0.046*
**31.25**	0.003**	0.042*	0.041*	0.047*
**15.62**	0.002**	0.048*	0.016*	0.048*
**7.8125**	0.021*	0.039*	0.025*	0.048*
**3.9**	0.033*	0.037*	0.029*	0.046*
**1.95**	0.028*	0.041*	0.041*	0.043*
**0.97**	0.037*	0.043*	0.044*	0.047*
**0.488**	0.016*	0.045*	0.034*	0.047*

***p*-values less than 0.05 are statistically significant. $^{*}p < 0.05$, $^{**}p < 0.01$, $^{***}p < 0.001$, ns: not significant.**

Table S4(h). Estimated *p*-value and significance levels of doxycyline against biofilm destruction by gram-negative and gram-positive bacteria at 48 h.

**Concentration** (µg/mL)	**Doxycyline**			
	**MSSA**	**MRSA**	***K. pneumoniae***	***A. baumannii***
**10000**	0.004**	0.002**	0.0001***	0.007**
**500**	0.004**	0.006**	0.0008***	0.008**
**250**	0.005**	0.007**	0.0008***	0.007**
**125**	0.012*	0.002**	0.003**	0.006**
**62.5**	0.025*	0.003**	0.004**	0.008**
**31.25**	0.020*	0.003**	0.004**	0.008**
**15.62**	0.018*	0.010*	0.014*	0.007**
**7.8125**	0.021*	0.002**	0.040*	0.006**
**3.9**	0.038*	0.003**	0.043*	0.005**
**1.95**	0.043*	0.007**	0.020*	0.041*
**0.97**	0.042*	0.042*	0.040*	0.007**
**0.488**	0.048*	0.041*	0.033*	0.007**

***p*-values less than 0.05 are statistically significant. $^{*}p < 0.05$, $^{**}p < 0.01$, $^{***}p < 0.001$, ns: not significant.**

Table S5. List of targets, PDB IDs, resolution, and active site coordinates.

NO	Protein Targets	Organisms	PDB ID	Resolution	Active site coordinates:			References
					X	Y	Z	
1	penicillin-binding proteins	***A. baumannii***	**3UE1**	2.73 Å	-33.2	-10.30	5.57	(1)
**2**	KPC-2 Carbapenemase	***K. pneumoniae***	**2OV5**	1.85 Å	54.45	-23.12	0.49	(2)
**3**	penicillin-binding proteins (PBP2a)	***Methicillin-resistant S. aureus***	**4CJN**	1.95 Å	13.20	-1.90	-70.10	(3)
**4**	penicillin-binding protein 3 (PBP3)	***Methicillin-sensitive S. aureus***	**3VSL**	2.40 Å	19.85	-49.55	22.28	(4)

References

1. Han S, Caspers N, Zaniewski RP, Lacey BM, Tomaras AP, Feng X, Geoghegan KF, Shanmugasundaram V. 2011. Distinctive attributes of β-lactam target proteins in Acinetobacter baumannii relevant to development of new antibiotics. J Am Chem Soc 133:20536–20545.

2. Ke W, Bethel CR, Thomson JM, Bonomo RA, van den Akker F. 2007. Crystal structure of KPC-2: insights into carbapenemase activity in class A beta-lactamases. Biochemistry 46:5732–5740.

3. Bouley R, Kumarasiri M, Peng Z, Otero LH, Song W, Suckow MA, Schroeder VA, Wolter WR, Lastochkin E, Antunes NT, Pi H, Vakulenko S, Hermoso JA, Chang M, Mobashery S. 2015. Discovery of antibiotic (E)-3-(3-carboxyphenyl)-2-(4-cyanostyryl)quinazolin-4(3H)-one. J Am Chem Soc 137:1738–1741.

4. Yoshida H, Kawai F, Obayashi E, Akashi S, Roper DI, Tame JRH, Park S-Y. 2012. Crystal structures of penicillin-binding protein 3 (PBP3) from methicillin-resistant Staphylococcus aureus in the apo and cefotaxime-bound forms. J Mol Biol 423:351–364.
